# Changes in problem-solving style when pain does not resolve. A longitudinal analysis of adults with chronic pain after total knee replacement

**DOI:** 10.1097/j.pain.0000000000003799

**Published:** 2025-09-09

**Authors:** Anna Gibby, Maya Braun, Wendy Bertram, Geert Crombez, Rachael Gooberman-Hill, Tim J. Peters, Vikki Wylde, Christopher Eccleston

**Affiliations:** a Centre for Pain Research, University of Bath, Bath, United Kingdom; b Department of Experimental-Clinical and Health Psychology, Ghent University, Ghent, Belgium; c Institue for Implementation Science in Healthcare, University of Zurich, Zurich, Switzerland; d Bristol Medical School, University of Bristol, Bristol, United Kingdom; e National Institute for Health Research, Bristol Biomedical Research Centre, Bristol, United Kingdom; f Bristol Dental School, University of Bristol, Bristol, United Kingdom; g Department of Psychology, The University of Helsinki, Helsinki, Finland

**Keywords:** Chronic pain, Total knee replacement, Problem solving, Acceptance

## Abstract

One-fifth of adults who receive a total knee replacement (TKR) go on to develop chronic pain. The behavioural approach taken to find a solution to pain may vary from assimilative (pursuit of analgesia) to accommodative (acceptance of pain insolubility and adoption of alternative goals). A total of 313 patients participated in a trial of an enhanced care pathway for TKR reported pain at 3 months after surgery. Participants undertook treatment as usual or treatment as usual with a new optimised support and treatment after replacement care pathway. Several pain and psychosocial outcomes (including problem-solving) were assessed. Two of the 3 problem-solving subscales moved from assimilative to accommodative over time (solving pain, acceptance of insolubility) and the overall belief in a solution decreased. Despite this shift to a more accommodative (accepting) problem-solving approach, participants reported lower meaningfulness of life over time. This reduced meaningfulness of life was associated with an increase in pain severity, interference, and reduced knee function. These interactions were not moderated by the treatment pathway to which participants were allocated. Overall, these results suggest that while patients who have pain after TKR become less determined to pursue analgesic solutions, this accommodation is not a naturally occurring positive acceptance. We discuss the possible reasons for this lack of functional and adaptive accommodation to chronic pain over the 12 months postoperative period.

## Abstract 2

Supplemental Digital Content is Available in the Text.

A secondary data analysis that explores the trajectory of problem-solving approaches to persistent pain after total knee replacement and how these affect chronic pain outcomes.

## 1. Introduction

Primary total knee replacement (TKR) is a common surgery undertaken to reduce pain and improve function, largely for people with osteoarthritis. One-fifth of patients report chronic pain postoperatively,^1^ and pain at 3 months predicts pain at 1 year.^15^ Nonetheless some people do recover from chronic postoperative pain.^8^ We established a research framework to emphasise factors that implicate recovery or maintenance of chronic pain, recognising that the mechanisms of onset are likely to be different from the mechanisms of maintenance.^13^ Prospective studies of the maintenance of chronic pain after TKR show that preoperative pain, negative affect, smoking, obesity, worry about pain, kinesiophobia and disrupted sleep are risk factors,^5,14,22,35^ but there are few empirical studies of how people behave in response to chronic pain after TKR. Qualitative studies indicate that people struggle with pain and other sensory changes (pressure, heaviness, swelling) and report a lack of agency with their limb, perceiving it as disembodied.^23^

When confronted with persistent pain, one can focus effort on finding a resolution to pain or learning to live with unresolved pain. This dynamic has been discussed as a “problem-solving” coping style^32^ and has been most explored within motivational theory in the context of normal ageing.^3,4^ An individual, confronted with a problem will choose a strategy. As they age, the problem may become chronic and it is important to understand how long one persists in seeking a particular solution.^30,32^ This approach to coping that focuses on seeking a solution to chronic pain can be measured using the pain solutions questionnaire (PaSol).^11^

We took the opportunity to investigate whether and how coping with chronic pain changes over time in a large randomised controlled trial (RCT) with adults with chronic pain after TKR. The RCT evaluated the clinical and cost-effectiveness of a new care pathway intervention (STAR), which had been designed to improve outcomes for those with pain at 3 months after TKR.^36^ The STAR RCT had 2 coprimary outcomes: self-reported pain severity and pain interference in the replaced knee, assessed with the Brief Pain Inventory (BPI) pain severity and interference scales at 12 months (scored 0-10, best to worst); a total of 313 patients (86%) provided these data (213 intervention plus usual care and 100 usual care alone). At 12 months, the mean between-group difference in the BPI severity score was −0·65 (95% confidence interval −1·17 to −0·13; *P* = 0·014) and that for the BPI interference score was −0·68 (−1·29 to −0·08; *P* = 0·026), both favouring the intervention.

We posed 3 related questions. First, does problem-solving change over time? We hypothesised that problem-solving would shift from a style characterised by efforts to find a solution, to a style characterised by acceptance. Second, did the STAR intervention have any effect on problem-solving style? Third, are the problem-solving styles we measured 3 months after the surgery related to outcomes at 9 months and 15 months after surgery?

## 2. Methods

### 2.1. Design

A planned secondary analysis of data from the STAR RCT.^36^ Data were collected through an unmasked, parallel group, pragmatic, superiority, RCT conducted in 8 National Health Service hospitals in the United Kingdom. Data were collected at 3 months after the TKR operation (time 1), 9 months after TKR (time 2), and 15 months after TKR (time 3). Ethics approval was obtained from the South West-Central Bristol Research Ethics Committee (16/SW/0154).

### 2.2. Participants and recruitment

A full description of recruitment methods is provided in the trial report.^36^ For the current analysis, the sample consisted of 313 adults aged 18 years or older who had undergone primary TKR due to osteoarthritis and reported pain at 3 months postoperation, defined as a score of 14 or lower on the pain component of the Oxford Knee Scale (OKS).^10^ A score of 14 or lower has been identified as the threshold at which an individual's health-related quality of life begins to deteriorate.^26^ Recruitment was undertaken over a 32-month period.

For the trial, participants were randomly allocated to receive either care as usual (CAU) or CAU together with the STAR care pathway. A 1:2 allocation ratio was used to facilitate a pragmatic assessment of clinical effectiveness.

### 2.3. Self-report questionnaires

The 12-item Oxford Knee Scale^10^ was used to assess knee pain and function. A higher score represents less pain and better function and a lower score represents more pain and less function.

Pain severity and interference were measured using the Brief Pain Inventory.^7^ The Severity scale consists of 4 items asking the participant about the severity of their pain in different circumstances over the past 24 hours. The higher the score, the greater the pain severity. The Interference scale is a 7-item scale that asks the participant how pain interferes in 7 areas of life. A higher score indicates a greater level of life interference because of pain.

Problem-solving was measured with the PaSol a 14-item questionnaire divided into 4 subscales (Meaningfulness of Life Despite Pain Scale, Solving Pain Scale, Acceptance of Insolubility of Pain Scale, and Belief in a Solution Scale).^11^ It is designed to measure an individual's approach to problem-solving on a spectrum of competing styles labelled as accommodative and assimilative.^3,32^ An accommodative style is characterised by an acceptance that the goal of pain relief cannot be reached necessitating a change of goal. An assimilative style is characterised by a recruitment of further resource in the repeated pursuit of pain relief. These styles are not dichotomous and exist on a spectrum from accommodative to assimilative problem-solving.

A problem-solving score is calculated by summing the scores of the Solving Pain Scale and the reverse scores of the Meaningfulness of Life Scale and Acceptance of Insolubility of Pain Scale. A high score would indicate a more assimilative problem-solving style and a low score indicates a more accommodative problem-solving style. The Belief in a Solution Scale is measured with the purpose of mediating the relationship between the PaSol score and other factors. We do not use it for this purpose in the current analysis but instead analyse the score descriptively and report how the score changes over time.^9^ The use of the PaSol in this study design is supported by content analysis by Lauwerier et al.^17^ which found that the PaSol was well-matched to their heuristic framework of acceptance.

### 2.4. Analysis plan

Data were processed and analysed in RStudio version 2023.12.1.402 (R Core Team, 2021). Scores were calculated for each subscale of the PaSol by creating a mean of the respective items.

For question 1 (whether problem-solving style changes between times 1, 2, and 3), linear multilevel models were fitted separately for each subscale of the PaSol, with Time as an independent variable. First, models with no random intercept were fitted. Then, models with a random intercept for each individual were fitted, and the models were compared. Finally, models with a random intercept for each individual and a random slope for time were fitted and compared with the models with only a random intercept. For each model, linearity and normal distribution of the residuals were investigated visually, and homogeneity of variance was investigated using Levene tests. Where the overall *P*-value for Time on a subscale was less than 5%, pairwise comparisons were conducted using paired t-tests (ie, time 1 vs time 2, time 1 vs time 3, time 2 vs time 3). *P*-values for these multiple comparisons are compared both with an uncorrected alpha level and with a corrected alpha level using Bonferroni correction.^2^

For question 2 (whether problem-solving styles differ by group over time), linear multilevel models were fitted in the same way as for research question 1, but with Time and Treatment Arm (CAU vs CAU plus STAR), as well as their interaction, included as explanatory variables.

For question 3 (whether problem-solving style at time 1 predicts outcomes at time 3), linear regression models were fitted for pain severity and pain interference as measured by the BPI^7^ as well as for knee-specific pain and physical function as measured by the OKS,^10^ at time 3. All subscales of the PaSol were included as explanatory variables, as well as the baseline score for the respective outcome measure. In exploratory analyses, interaction effects of trial arm (CAU vs CAU plus STAR) and the subscales of the PaSoL were added to the model in an additional step.

In further exploratory analyses, we aimed to understand whether patients could be clustered based on their problem-solving style at time 1. More information on both the analysis used and the resulting clusters is provided in Appendix 1, http://links.lww.com/PAIN/C378.

This study and analysis plan were registered before analysis at Open Science Framework (https://osf.io/4t6pd/). However, we deviated from the originally registered plan in multiple important ways. First, we decided to use multilevel linear regression for research questions 1 and 2 to account for the structure of the data set more appropriately. Second, we focused on the specific subscales of the PaSol, rather than the total sum score, throughout analyses. Finally, we broadened the outcomes considered in research question 3 to include pain severity, interference, and physical function, rather than focusing solely on pain.

## 3. Results

### 3.1. Descriptive statistics on sample and outcomes

Table 1 summarises all descriptive data on pain severity and interference using the Oxford Knee Score and the Brief Pain Inventory. At time 1, the OKS was severe with a mean of 18.23 improving to moderate at 25.72 at time 2, and 28.03 at time 3. The pain similarly improved from time 1 to time 2 and time 3 in both severity and interference: Severity improved over time from 5.24 at time 1 to 3.71 at time 2, and 3.31 at time 3. Interference improved over time from 6.28 at time 1 to 4 at time 2, and 3.70 at time 3. Overall, 85.9% of participants reported chronic pain at time 3, with a score of 14 or less on the OKS.

**Table 1:** Descriptive statistics for problem-solving styles and pain outcomes.

Variable	Time point	Min	Q1	Median	Q3	Max	Mean	SD
Belief in a solution	Time 1	1.0	4.0	5.5	7.0	7.0	5.29	1.56
	Time 2	1.0	3.0	4.5	6.0	7.0	4.37	1.94
	Time 3	1.0	1.5	4.0	6.0	7.0	3.9	2.17
Solving pain scale	Time 1	1.0	4.5	5.5	6.5	7.0	5.29	1.46
	Time 2	1.0	3.0	4.75	6.0	7.0	4.46	1.9
	Time 3	1.0	2.5	4.62	6.0	7.0	4.21	2.01
Acceptance of insolubility of pain	Time 1	1.0	2.67	3.67	4.67	7.0	3.63	1.58
	Time 2	1.0	3.0	4.0	5.33	7.0	3.95	1.78
	Time 3	1.0	2.33	4.0	5.33	7.0	3.85	1.89
Meaningfulness of life despite pain	Time 1	1.0	4.6	5.4	6.2	7.0	5.34	1.19
	Time 2	1.0	4.2	5.4	6.2	7.0	5.04	1.59
	Time 3	1.0	4.0	5.2	6.2	7.0	4.88	1.74
OKS	Time 1	3.0	14.0	19.0	22.0	32.0	18.23	5.83
	Time 2	4.0	19.0	26.0	33.0	46.0	25.72	9.28
	Time 3	5.0	20.0	28.5	36.0	48.0	28.03	10.07
BPI severity	Time 1	0.5	4.0	5.25	6.5	10.0	5.24	1.69
	Time 2	0.0	1.81	3.38	5.25	9.75	3.71	2.37
	Time 3	0.0	1.0	3.0	5.0	10.0	3.31	2.47
BPI interference	Time 1	1.57	5.0	6.43	7.71	10.0	6.28	1.91
	Time 2	0.0	1.86	4.0	6.43	9.86	4.15	2.66
	Time 3	0.0	1.14	3.29	6.0	9.86	3.7	2.83

BPI, Brief Pain Inventory; OKS, Oxford Knee Scale.

### 3.2. Does problem-solving style change over time?

Overall patients became more accommodative from time 1 to time 2 and time 2 to time 3.

Table 2 summarises all the pairwise comparisons for PaSol subscales at different measurement occasions. Time was associated with the outcomes for each subscale of the PaSol, with low *P*-values for virtually all pairwise comparisons between the 3 time points. Scores on the Solving Pain Scale and Belief in a Solution Scale decreased between time 1 and time 3, translating as a more accommodative score. Acceptance of the Insolubility of Pain increases between time 1 and time 3, again translating to more accommodative score. Score on the Meaningfulness of Life decreased between time 1 and time 3 translating to a more assimilative score. The Effect Sizes differed and were largest between time 1 to time 2 for the subscales Acceptance of Insolubility of Pain (*d* = 0.59) and Solving Pain (*d* = 0.50). The direction of the change was negative, with mean values decreasing from earlier to later time points for all subscales except for Acceptance of the Insolubility of Pain, which increased from time 1 to time 2.

**Table 2:** Pairwise comparison for pain solutions questionnaire subscales at different measurement occasions.

Comparison	Subscale	Mean difference	t	Cohen's d	df	*P*	95% CI lower	95% CI upper
Time 1 − Time 2	Acceptance of insolubility	0.38	3.16	0.18	291	0.002	−0.62	−0.14
	Meaningfulness despite pain	−0.31	−2.91	0.17	298	0.004	0.1	0.53
	Belief in a solution	−0.94	−7.25	0.42	294	<0.001	0.69	1.2
	Solving pain	−0.78	−6.85	0.4	297	<0.001	0.56	1.01
Time 1 − Time 3	Acceptance of insolubility	0.31	2.44	0.15	275	0.015	−0.57	−0.06
	Meaningfulness despite pain	−0.53	−4.66	0.28	282	<0.001	0.31	0.76
	Belief in a solution	−1.43	−9.87	0.59	280	<0.001	1.15	1.72
	Solving pain	−1.05	−8.34	0.5	281	<0.001	0.8	1.3
Time 2 − Time 3	Acceptance of insolubility	−0.13	−1.08	0.07	255	0.28	−0.11	0.37
	Meaningfulness despite pain	−0.27	−2.42	0.15	261	0.016	0.05	0.48
	Belief in a solution	−0.58	−4.14	0.26	259	<0.001	0.3	0.85
	Solving pain	−0.35	−2.89	0.18	260	0.004	0.11	0.58

95% CI, 95% confidence interval.

Post hoc, we determined that meaningfulness of life and depression were negatively correlated. This correlation was strongest at time 1 (−0.40), decreased at time 2 (−0.23) and time 3 (−0.11).

### 3.3. Does problem-solving style differ by group over time?

Problem-solving style did not differ between the CAU and the CAU plus STAR groups over time for any subscale at any time point (Table 3). Consequently, no pairwise comparisons were performed.

**Table 3:** Overview of multilevel linear regressions aiming to predict different subscales of the pain solutions questionnaire.

		df	*P*
Time	Solving pain	1	<0.001
	Meaningfulness of life despite pain	1	<0.001
	Acceptance of pain	1	0.028
	Belief in insolubility of pain	1	<0.001
Treatment arm	Solving pain	1	0.699
	Meaningfulness of life despite pain	1	0.763
	Acceptance of pain	1	0.699
	Belief in insolubility of pain	1	0.822
Time × treatment	Solving pain	1	0.301
	Meaningfulness of life despite pain	1	0.568
	Acceptance of pain	1	0.675
	Belief in insolubility of pain	1	0.626

### 3.4. Does problem-solving style at time 1 predict pain outcomes at time 3?

As summarised in Table 4, the subscale Meaningfulness of Life Despite Pain predicted all outcomes, while Acceptance of Insolubility of Pain only predicted BPI Interference and OKS scores. Belief in a Solution and the Solving Pain subscales are not predictive of any outcomes.

**Table 4:** Overview of linear regressions aiming to predict outcomes at time 3 using time 1 characteristics.

	R^2^	SE	*P*
Predicting severity at time 3 using time 1 characteristics			
BL severity	0.641	0.081	<0.001
Acceptance	0.092	0.084	0.276
Belief	−0.036	0.099	0.715
Meaning	−0.300	0.135	0.027
Solve	0.035	0.093	0.709
Predicting pain interference at time 3 using time 1 characteristics			
BL interference	0.552	0.082	<0.001
Acceptance	0.211	0.095	0.026
Belief	−0.044	0.113	0.699
Meaning	−0.424	0.152	0.006
Solve	0.188	0.111	0.091
Predicting OKS at time 3 using time 1 characteristics			
BL OKS	0.818	0.094	<0.001
Acceptance	−0.733	0.336	0.030
Belief	−0.178	0.399	0.655
Meaning	1.649	0.539	0.003
Solve	−0.276	0.372	0.460

BL, Baseline; OKS, Oxford Knee Scale.

## 4. Discussion

Three hundred thirteen adults with osteoarthritis of the knee reported chronic pain after total knee replacement surgery. At 3 months after the surgery, they reported pain and interference. At 3 months, their problem-solving style was largely assimilative, meaning they believed that a solution for their pain could be found and efforts could usefully be made to remove or attenuate the pain and its negative consequences. Nine months after surgery, with pain persisting for most of the participants, they had become less assimilative, more accommodative, and less likely to believe that a solution could be found. Further analysis of the subscales of the PaSol measure, however, revealed a more complex picture. Although the sum PaSol score and the individual subscores changed overtime, the individual subscores operated in different directions. Patients were less likely to believe that a solution for their pain could be found (subscales Solving Pain, Belief in a Solution and Acceptance of Insolubility of Pain), but this acceptance was not supported by the subscale Meaningfulness of Life Despite Pain, which was also found to be correlated inversely with depression. In short, this accommodative coping style is not necessarily a path to positive outcomes, namely, increased quality of the life and improved mood. Furthermore, the shift from an assimilative to an accommodative style was not a result of the STAR treatment pathway. Higher pain interference and the lower overall knee function 9 months after surgery were better predicted by a lower meaningfulness of life and greater acceptance of the insolubility of pain at 3 months after surgery. Increased pain severity was also predicted by lower meaningfulness of life 9 months after surgery.

In behaviour therapy, acceptance of pain has a specific meaning: It refers to a largely positive adaptive state in which one is able to separate pain from suffering and adopt a willingness to experience discomfort without sacrificing engagement with meaningful life activities.^21^ There is a large literature on psychological interventions which aims through direct intervention to facilitate this outcome.^34^ However, as with other psychological constructs, the use of nontechnical language and common terms for specific processes can cause confusion. For patients, acceptance can mean anything from a positive state of “letting go and refocusing” to a negative experience of “surrender or abandonment.”^28^

This linguistic challenge is relevant here. We chose to stay close to the labels used in the PaSol in our description of the findings. However, the analysis revealed an interesting finding: overall there seems to be a shift from an assimilative style to a more accommodative style of problem-solving, in which one no longer pursues pain relief. However, this shift is not therapeutic or adaptive. It is not the sort of acceptance discussed as a positive state in acceptance and commitment therapy. Instead, it is better thought of as a giving up of an unhelpful negative strategy but replacing it with an attitude of negative realism that any positive change may not be possible, of not expecting or pursuing positive meaningful life outcomes.

There is no intrinsic value of a particular problem-solving style—its value is contextual and often justified by outcome.^31,32^ A pursuit of pain relief, exploring all options, seeking further opinions, and attempting different analgesic solutions, may be functional and relevant in a postoperative environment. Similarly, accepting pain too early may be dysfunctional. This intriguing effect of changing problem-solving approach could be a function of time or reflect the lack of any intervention content aimed to facilitate acceptance.

We were only able to measure problem-solving style from 3 months up to 15 months after surgery and no preoperative data were collected. We do not know whether this process of relinquishing attempts to achieve analgesia and adopting a focus on achievable nonanalgesic goals takes more time or whether the switching between styles described as a futility loop is part of an adaptive process.^24^ The lack of meaning may be part of a loss or depressive adaptation.^25^ For example, it is possible that beliefs about the insolubility of pain are core, change first, and lead to a judgement of the rewards of life as having meaning. Early adaptation may be dominated by the loss of unachievable goals, goals not yet replaced by new goals which offer new meaning in life.^12^ We know very little about the temporality or dynamics of that process. Furthermore, we do not know the role of the preoperative chronic pain the participants were likely to have lived with for many years before the surgery. In addition, we have not been able to include in our analysis previously established mechanisms of postoperative pain outcomes that occur preoperatively, such as preoperative pain severity and negative affect.^14^ Individuals' problem-solving for their knee pain likely started long before surgery. Much of the literature on chronic pain and acceptance is with chronic pain patients with much longer histories of pain, disability, and depression, in those who are stuck in a constant state of high impact pain.^20^ Similarly, this early sign of loss of meaning may be prodromal for a more rigid presentation of high impact, treatment-resistant chronic pain.^29^

The extent to which the switching away from the goal of pain relief is acceptable is debatable. Unlike other life goals lost and replaced, health goals and in particular the relief of suffering should be considered a special class. There is no reason to believe that one can reach a healthy acceptance of a life lived in persistent pain without psychotherapeutic intervention. Moreover, considering that this is a population who were actively seeking an analgesic solution, so we might expect those capable of a natural shift to acceptance to have not selected surgery, noting that the STAR intervention had no specific content that could bring about this change. Chronic pain is also a social phenomenon, its deleterious effects are personally significant and stubborn but it also affects others.^18,27^

In addition, we prioritise a dynamic process of problem-solving, but have only 3 data points per individual. Furthermore, although we have a large sample, we are still limited to our planned analyses by statistical power.

Future studies could focus usefully on several areas. First, the extent to which this emergence of a growing belief in the insolubility of pain coupled with a loss of meaning in life is either a temporary adaptive response to loss or a risk factor for later stubborn high impact chronic pain should be investigated. Second, there is a common finding in this field that expectations of a positive outcome can act as a placebo.^16^ It is also possible that the effect of heightened expectations when they are unmet make the switching from a problem-solving style focussed on analgesia to one focussed on acceptance more difficult.^6^ Third, there is an opportunity to target adaptive problem-solving with psychotherapeutic content aimed at improving acceptance of pain: the exact content, timing, duration, and format are open questions. Finally, the extent to which these findings are general to the onset and maintenance of chronic pain is interesting. Orthopaedic surgery is an excellent human model for investigating psychological processes, given the large and growing number of people who will undergo surgery^19^ and the relative standardisation of the peri-operative process.

In conclusion, we now have some understanding of how problem-solving style may change over time for adults with chronic pain after knee replacement surgery—specifically, patients generally become more accommodating in response to their pain. However, this accommodation is not a therapeutic acceptance, because the ability to find life meaningful worsens and is associated with poorer outcomes. It is tempting to believe that people simply adjust over time, or that self-management can be achieved through education or low-intensity supportive interventions. The evidence suggests otherwise. Successfully accommodating to pain needs expert psychological pain management.^33^ Recognising that acceptance does not emerge naturally following 1 year with chronic pain after surgery should focus our attention on preventative or therapeutic interventions to enhance adaptive problem-solving, preemptively before surgery, or early on when pain does not resolve.

## Conflict of interest statement

The authors have no conflicts of interest to declare.

## Supplementary Material

## Appendix A. Supplemental digital content

Supplemental digital content associated with this article can be found online at http://links.lww.com/PAIN/C378.
