# Supplementary material for: Changes in problem-solving style when pain does not resolve. A longitudinal analysis of adults with chronic pain after total knee replacement
Source: Pain. 2025 Sep 9;167(2):338–43. doi: 10.1097/j.pain.0000000000003799 (PMC12794342; doi:10.1097/j.pain.0000000000003799)
Supplement: Supplementary file 1 [file jop-167-338-s001.pdf]

Analysis STAR

Maya Braun

2025-01-27

Descriptive Analyses

```
table(star_wide$ou_arm)
```

```
##
##   1   2
## 242 121
```

```
table(star_wide$Gender)
```

```
##
##   F   M
## 217 146
```

```
summary(star_wide$Age)
```

```
##    Min. 1st Qu.  Median    Mean 3rd Qu.    Max.    NA's
##   40.00   61.11   67.00   67.13   73.00   88.28       1
```

```
variables <- c("pa_sol_belief_BL", "pa_sol_solve_BL", "pa_sol_accept_BL", "pa_sol_meaning_BL",
               "oks_BL", "bpi_severity_BL", "bpi_int_BL",
               "pa_sol_belief_FU1", "pa_sol_solve_FU1", "pa_sol_accept_FU1", "pa_sol_meaning_FU
1",
               "oks_FU1", "bpi_severity_FU1", "bpi_int_FU1",
               "pa_sol_belief_FU2", "pa_sol_solve_FU2", "pa_sol_accept_FU2", "pa_sol_meaning_FU
2",
               "oks_FU2", "bpi_severity_FU2", "bpi_int_FU2")

# Function to get summary including standard deviation
get_summary <- function(var) {
  data <- star_wide[[var]]
  summary_stats <- quantile(data, probs = c(0, 0.25, 0.5, 0.75, 1), na.rm = TRUE)
  names(summary_stats) <- c("Min", "1st Qu.", "Median", "3rd Qu.", "Max")
  mean_value <- mean(data, na.rm = TRUE)
  sd_value <- sd(data, na.rm = TRUE)
  return(c(summary_stats, "Mean" = mean_value, "Std.Dev" = sd_value))
}

# Apply the function to each variable and store the results
results <- round(sapply(variables, get_summary),2)

# Convert the results to a data frame for better readability
summary_df <- as.data.frame(t(as.data.frame(results)))
summary_df$Variable <- rownames(summary_df)
print(summary_df)
```

```
##                      Min 1st Qu. Median 3rd Qu.   Max  Mean Std.Dev
## pa_sol_belief_BL   1.00    4.00   5.50    7.00  7.00  5.29    1.56
## pa_sol_solve_BL    1.00    4.50   5.50    6.50  7.00  5.29    1.46
## pa_sol_accept_BL   1.00    2.67   3.67    4.67  7.00  3.63    1.58
## pa_sol_meaning_BL  1.00    4.60   5.40    6.20  7.00  5.34    1.19
## oks_BL             3.00   14.00  19.00   22.00 32.00 18.23    5.83
## bpi_severity_BL    0.50    4.00   5.25    6.50 10.00  5.24    1.69
## bpi_int_BL         1.57    5.00   6.43    7.71 10.00  6.28    1.91
## pa_sol_belief_FU1  1.00    3.00   4.50    6.00  7.00  4.37    1.94
## pa_sol_solve_FU1   1.00    3.00   4.75    6.00  7.00  4.46    1.90
## pa_sol_accept_FU1  1.00    3.00   4.00    5.33  7.00  3.95    1.78
## pa_sol_meaning_FU1 1.00    4.20   5.40    6.20  7.00  5.04    1.59
## oks_FU1            4.00   19.00  26.00   33.00 46.00 25.72    9.28
## bpi_severity_FU1   0.00    1.81   3.38    5.25  9.75  3.71    2.37
## bpi_int_FU1        0.00    1.86   4.00    6.43  9.86  4.15    2.66
## pa_sol_belief_FU2  1.00    1.50   4.00    6.00  7.00  3.90    2.17
## pa_sol_solve_FU2   1.00    2.50   4.62    6.00  7.00  4.21    2.01
## pa_sol_accept_FU2  1.00    2.33   4.00    5.33  7.00  3.85    1.89
## pa_sol_meaning_FU2 1.00    4.00   5.20    6.20  7.00  4.88    1.74
## oks_FU2            5.00   20.00  28.50   36.00 48.00 28.03   10.07
## bpi_severity_FU2   0.00    1.00   3.00    5.00 10.00  3.31    2.47
## bpi_int_FU2        0.00    1.14   3.29    6.00  9.86  3.70    2.83
##                              Variable
## pa_sol_belief_BL     pa_sol_belief_BL
## pa_sol_solve_BL       pa_sol_solve_BL
## pa_sol_accept_BL     pa_sol_accept_BL
## pa_sol_meaning_BL   pa_sol_meaning_BL
## oks_BL                         oks_BL
## bpi_severity_BL       bpi_severity_BL
## bpi_int_BL                 bpi_int_BL
## pa_sol_belief_FU1   pa_sol_belief_FU1
## pa_sol_solve_FU1     pa_sol_solve_FU1
## pa_sol_accept_FU1   pa_sol_accept_FU1
## pa_sol_meaning_FU1 pa_sol_meaning_FU1
## oks_FU1                       oks_FU1
## bpi_severity_FU1     bpi_severity_FU1
## bpi_int_FU1               bpi_int_FU1
## pa_sol_belief_FU2   pa_sol_belief_FU2
## pa_sol_solve_FU2     pa_sol_solve_FU2
## pa_sol_accept_FU2   pa_sol_accept_FU2
## pa_sol_meaning_FU2 pa_sol_meaning_FU2
## oks_FU2                       oks_FU2
## bpi_severity_FU2     bpi_severity_FU2
## bpi_int_FU2               bpi_int_FU2
```

```
# write_xlsx(summary_df, "descriptives.xlsx")
```

Descriptive analysis: correlations

We are calculating correlations between all subscales and outcomes (throughout time)

```
subscales = c("pa_sol_solve", "pa_sol_meaning", "pa_sol_belief", "pa_sol_accept")
outcomes = c("bpi_severity", "bpi_int", "oks")


corrplot(cor(star[,c(subscales, outcomes)], use = "pairwise.complete.obs"), method = "number")
```

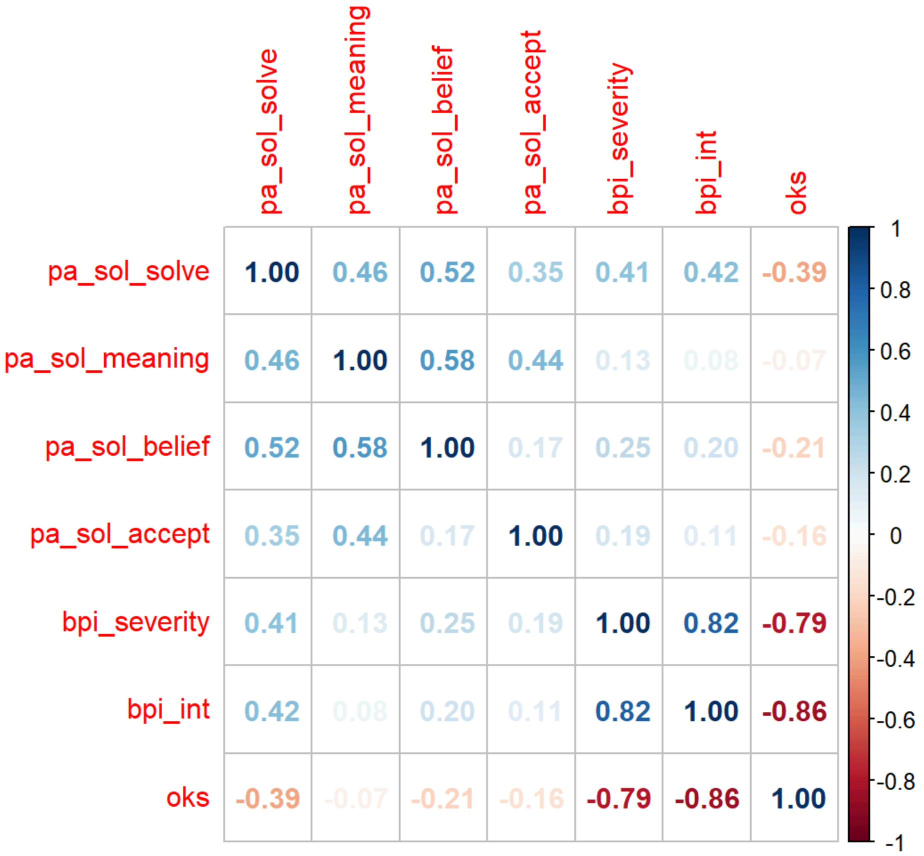

Research Question 1: Do adults who receive a total knee replacement due to osteoarthritis change their pain problem solving style between 3 months and 15 months post-operation?

Visualising question

We visualize a selection of the participants to get a better idea of the data.

```
sample <- sample(complete_cases, 50)
star_sample <- star[star$study_id %in% sample,]

star_gg2 <- star_sample %>%
  group_by(stage) %>%
  summarise(pa_sol = mean(pa_sol, na.rm=T))

theme_set(theme_minimal())

p = ggplot()+
  geom_line(data = star_sample, aes(stage, pa_sol, group = as.factor(study_id)), color= "grey",
show.legend =FALSE)
q = p + geom_line(data=star_gg2, aes(stage, pa_sol),color = "red", linewidth = 1)

print(q + ylim(1,7))
```

```
## Warning: Removed 3 rows containing missing values (`geom_line()`).
```

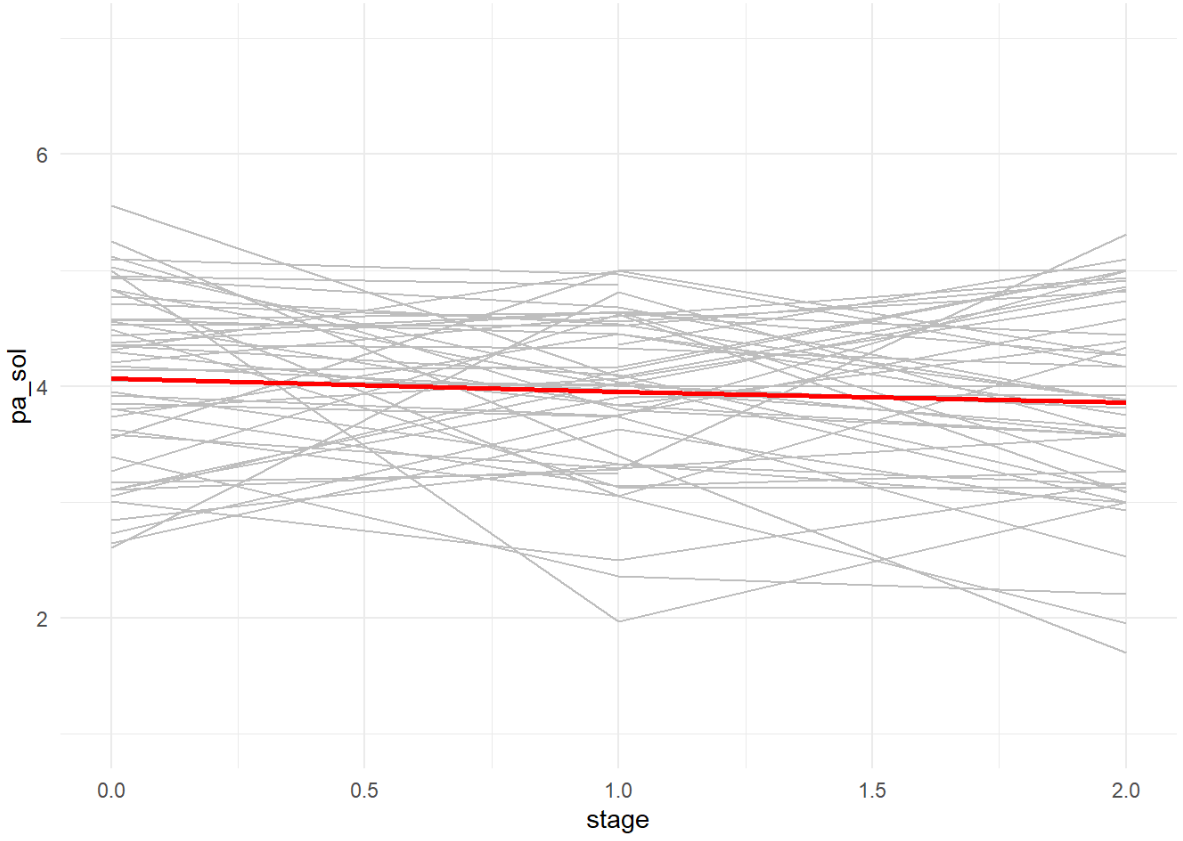


```
print(q + ylim(2,4.2))
```

```
## Warning: Removed 63 rows containing missing values (`geom_line()`).
```

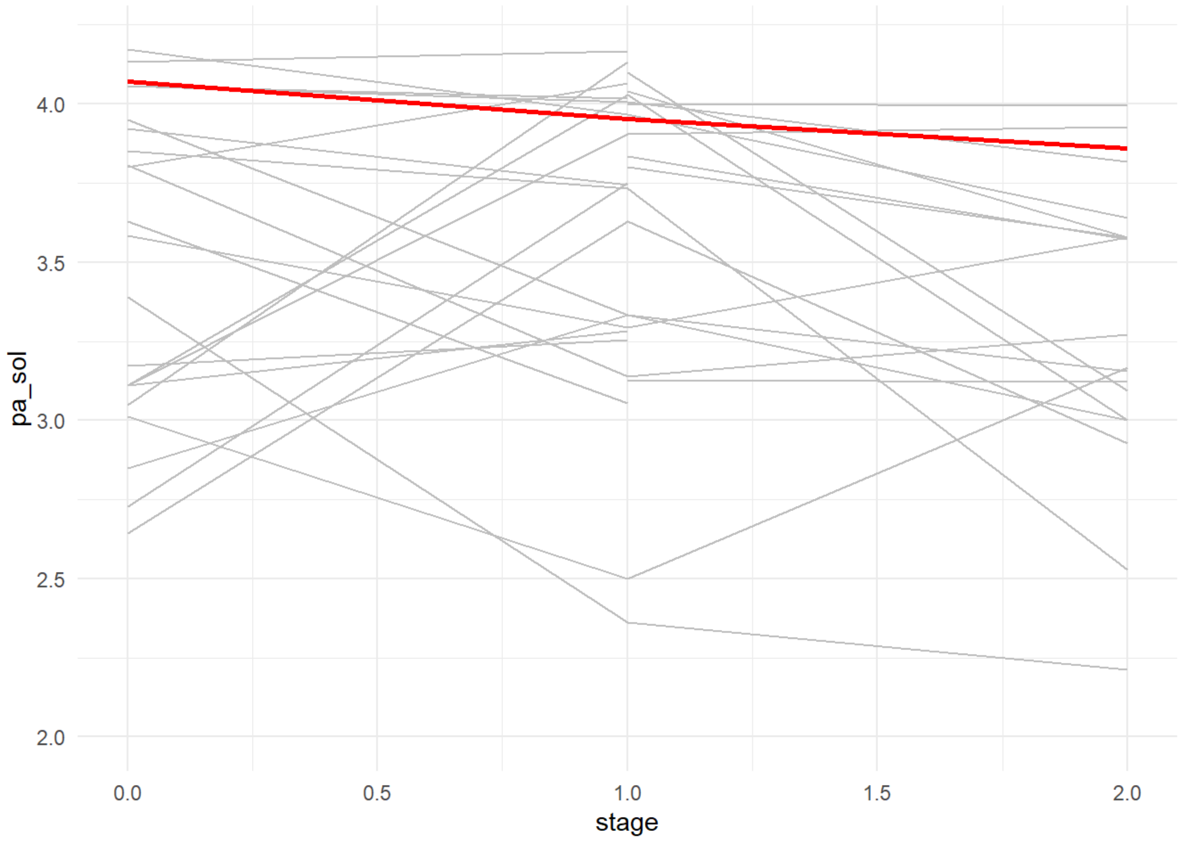

```
star_gg1 <- star %>%
  group_by(stage) %>%
  summarise(pa_sol_accept = mean(pa_sol_accept, na.rm=T),
            pa_sol_belief = mean(pa_sol_belief, na.rm=T),
            pa_sol_solve = mean(pa_sol_solve, na.rm= T),
            pa_sol_meaning = mean(pa_sol_meaning, na.rm=T),
            pa_sol = mean(pa_sol, na.rm=T))


legend <- gather(star_gg1, key = scale, value = value, c("pa_sol_accept", "pa_sol_belief", "pa_s
ol_solve", "pa_sol_meaning", "pa_sol"))

theme_set(theme_minimal())

p <- ggplot(legend, aes(x=stage, y=value, group = scale, colour = scale)) +
  geom_line(size = 1.2) +
  geom_point(shape = 15) + theme(legend.position="bottom")
```

```
## Warning: Using `size` aesthetic for lines was deprecated in ggplot2 3.4.0.
## ℹ Please use `linewidth` instead.
## This warning is displayed once every 8 hours.
## Call `lifecycle::last_lifecycle_warnings()` to see where this warning was
## generated.
```

```
print(p +
      scale_x_continuous(breaks= c(0,1,2), limits= c(0,2))+
      scale_y_continuous(breaks=c(1,2,3,4,5,6,7), limits=c(1,7))+
      guides(color = guide_legend(nrow = 3))
      )
```

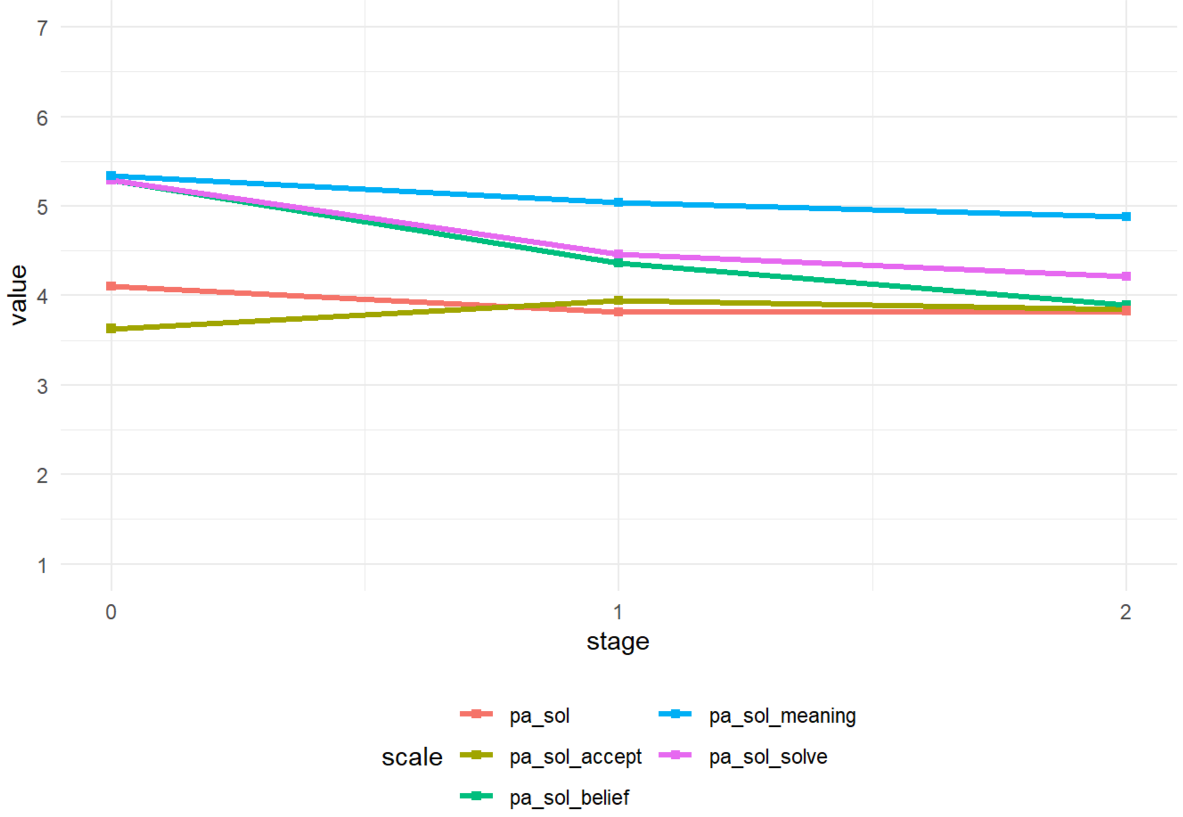

RQ1: Subscale Accept

```
model_accept0 <- lmer(pa_sol_accept ~ stage + (1| study_id), data = star)
model_accept1 <- lmer(pa_sol_accept ~ stage + (1 + stage | study_id), data = star, REML=FALSE)

anova(model_accept0, model_accept1)
```

```
## refitting model(s) with ML (instead of REML)
```

	npar <dbl>	AIC <dbl>	BIC <dbl>	logLik <dbl>	deviance <dbl>	Chisq <dbl>	Df <dbl>	Pr(>Chisq) <dbl>
model_accept0	4	3616.054	3635.404	-1804.027	3608.054	*NA*	*NA*	*NA*
model_accept1	6	3601.934	3630.958	-1794.967	3589.934	18.12088	2	0.0001161716

2 rows

```
Anova(model_accept1)
```

	Chisq <dbl>	Df <dbl>	Pr(>Chisq) <dbl>
stage	4.841375	1	0.02778473

1 row

```
summary(model_accept1)
```

```
## Linear mixed model fit by maximum likelihood  ['lmerMod']
## Formula: pa_sol_accept ~ stage + (1 + stage | study_id)
##    Data: star
##
##      AIC      BIC   logLik deviance df.resid
##   3601.9   3631.0  -1795.0   3589.9      926
##
## Scaled residuals:
##      Min       1Q   Median       3Q      Max
## -2.34891 -0.61753 -0.01244  0.60547  2.69516
##
## Random effects:
##  Groups   Name        Variance Std.Dev. Corr
##  study_id (Intercept) 0.5445   0.7379
##           stage       0.1467   0.3830   0.63
##  Residual             1.9589   1.3996
## Number of obs: 932, groups:  study_id, 362
##
## Fixed effects:
##             Estimate Std. Error t value
## (Intercept)  3.68960    0.07897   46.72
## stage        0.13530    0.06149    2.20
##
## Correlation of Fixed Effects:
##       (Intr)
## stage -0.489
```

```
# Linearity
plot(resid(model_accept1), main="Testing linearity of the model")
```

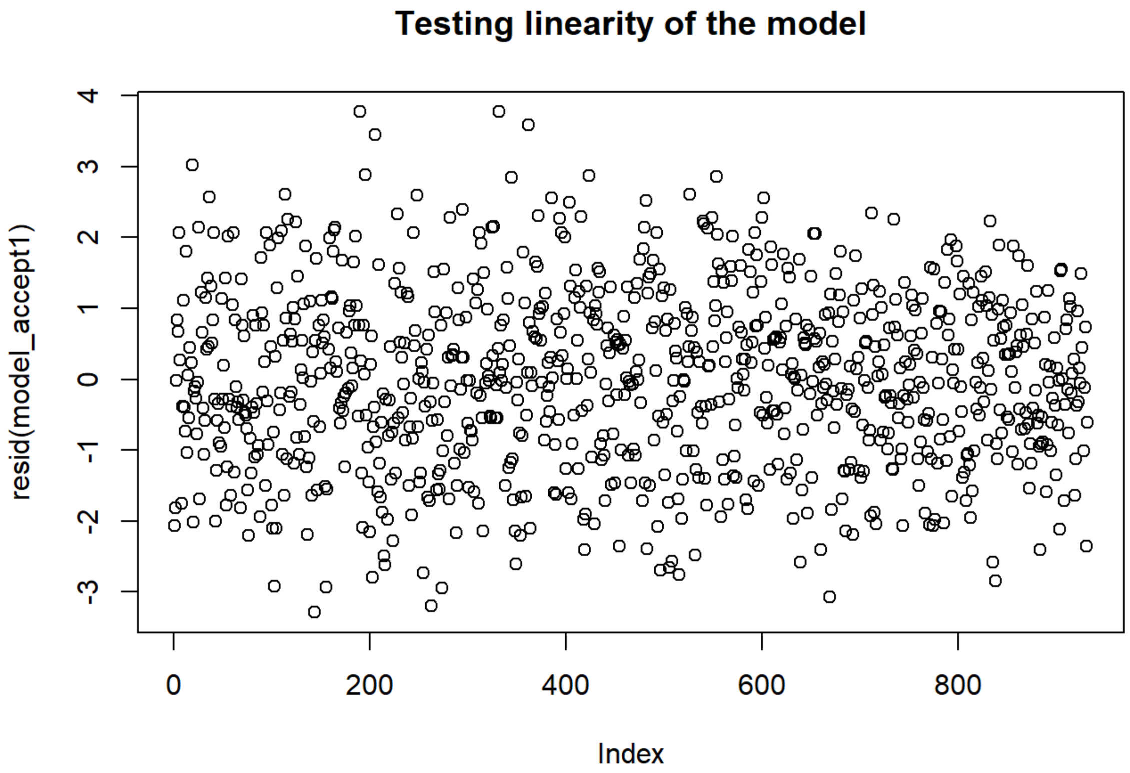


```
# Homogeneity of Variance: Levene Test
star1 <- star[!is.na(star$pa_sol_accept),]
star1$res2 <- abs(residuals(model_accept1))^2

anova(lm(star1$res2 ~ study_id, data = star1))
```

	Df <int>	Sum Sq <dbl>	Mean Sq <dbl>	F value <dbl>	Pr(>F) <dbl>
study_id	1	0.6870437	0.6870437	0.1769279	0.6741239
Residuals	930	3611.3616301	3.8831845	NA	NA

2 rows

```
# Normal distribution of residuals

qqmath(model_accept1, id = 0.05)
```

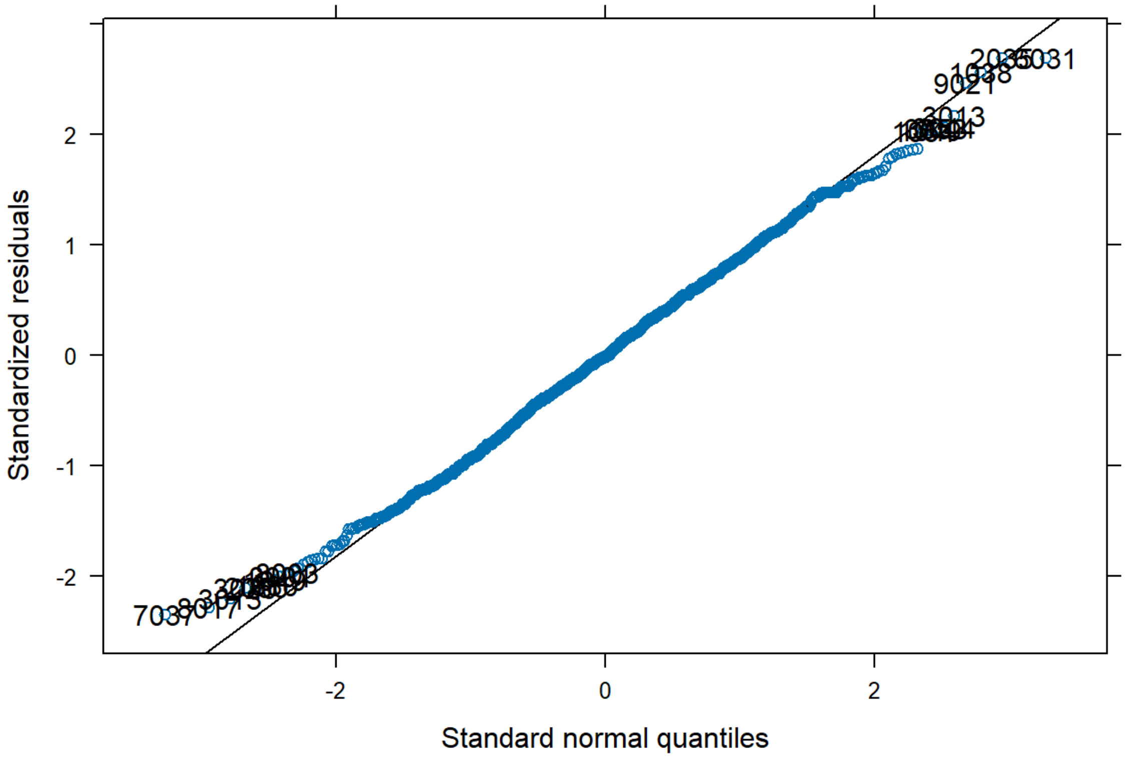


```
hist(residuals(model_accept1))
```

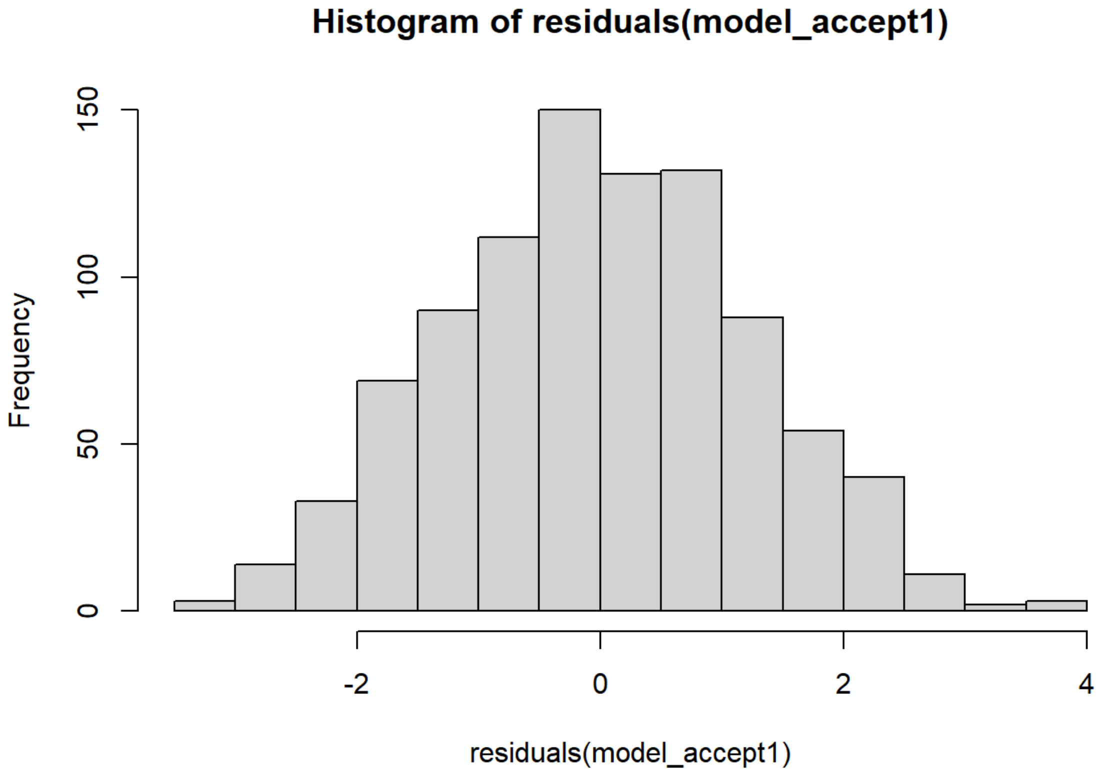

RQ1: Subscale Belief

```
model_belief0 <- lmer(pa_sol_belief ~ stage + (1| study_id), data = star)
model_belief1 <- lmer(pa_sol_belief ~ stage + (1 + stage | study_id), data = star)
```

```
## boundary (singular) fit: see help('isSingular')
```

```
anova(model_belief0, model_belief1)
```

```
## refitting model(s) with ML (instead of REML)
```

	npar<br><dbl>	AIC<br><dbl>	BIC<br><dbl>	logLik<br><dbl>	deviance<br><dbl>	Chisq<br><dbl>	Df<br><dbl>	Pr(>Chisq)<br><dbl>
model_belief0	4	3809.386	3828.770	-1900.693	3801.386	NA	NA	NA
model_belief1	6	3771.701	3800.776	-1879.851	3759.701	41.68517	2	8.875255e-10

2 rows

```
Anova(model_belief1)
```

	Chisq<br><dbl>	Df<br><dbl>	Pr(>Chisq)<br><dbl>
stage	104.8751	1	1.300832e-24

1 row

```
# Linearity
plot(resid(model_belief1), main="Testing linearity of the model")
```

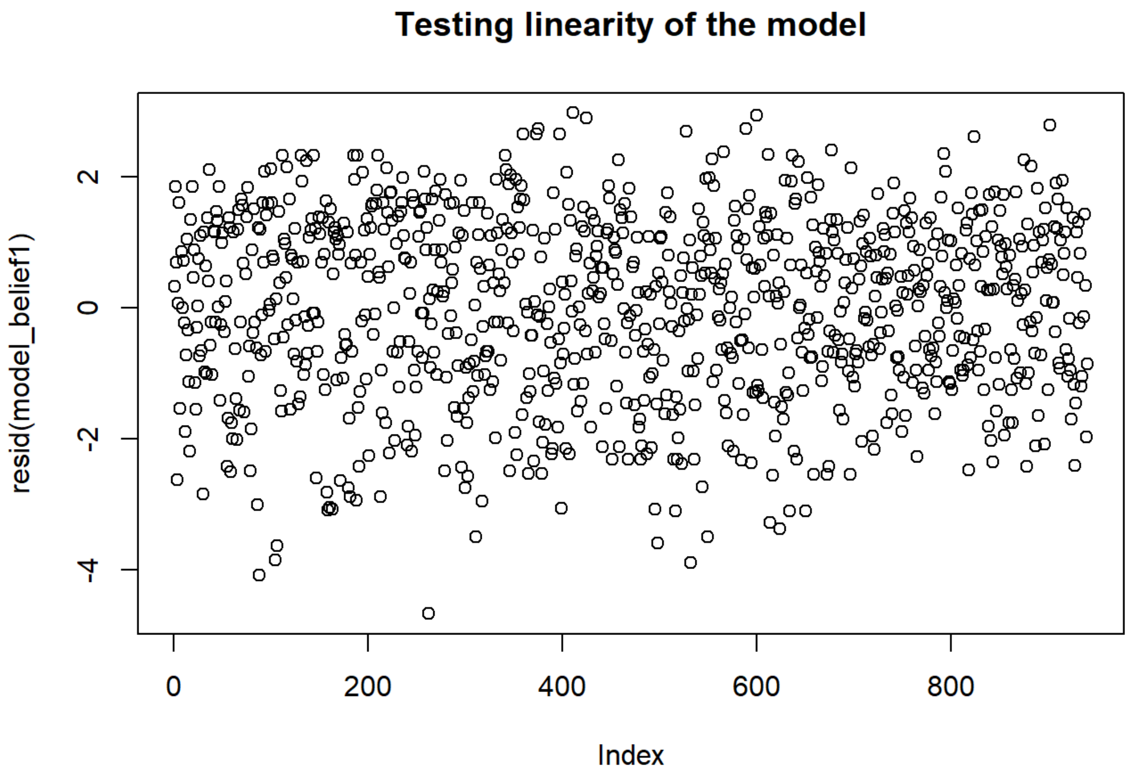


```
# Homogeneity of Variance: Levene Test
star1 <- star[!is.na(star$pa_sol_belief),]
star1$res2 <- abs(residuals(model_belief1))^2

anova(lm(star1$res2 ~ study_id, data = star1))
```

	Df<br><int>	Sum Sq<br><dbl>	Mean Sq<br><dbl>	F value<br><dbl>	Pr(>F)<br><dbl>
study_id	1	8.475199	8.475199	1.517599	0.218292
Residuals	938	5238.365609	5.584612	NA	NA

2 rows

```
# Normal distribution of residuals

qqmath(model_belief1, id = 0.05)
```

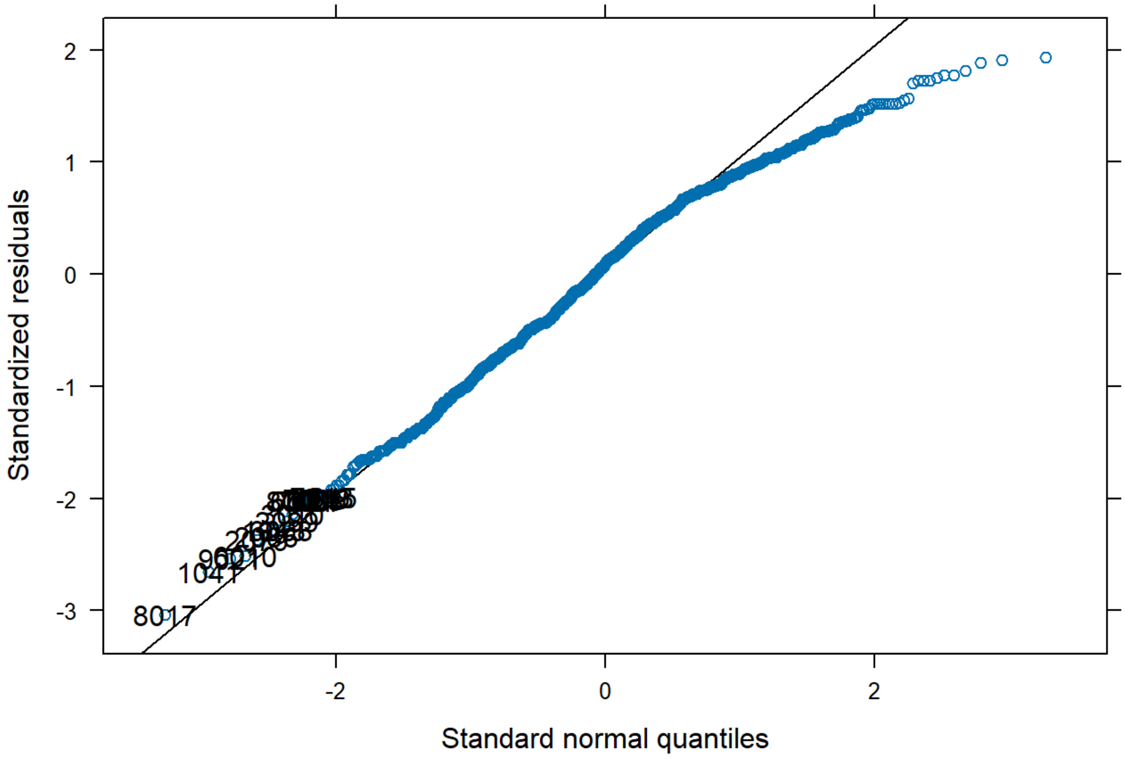


```
hist(residuals(model_belief1))
```

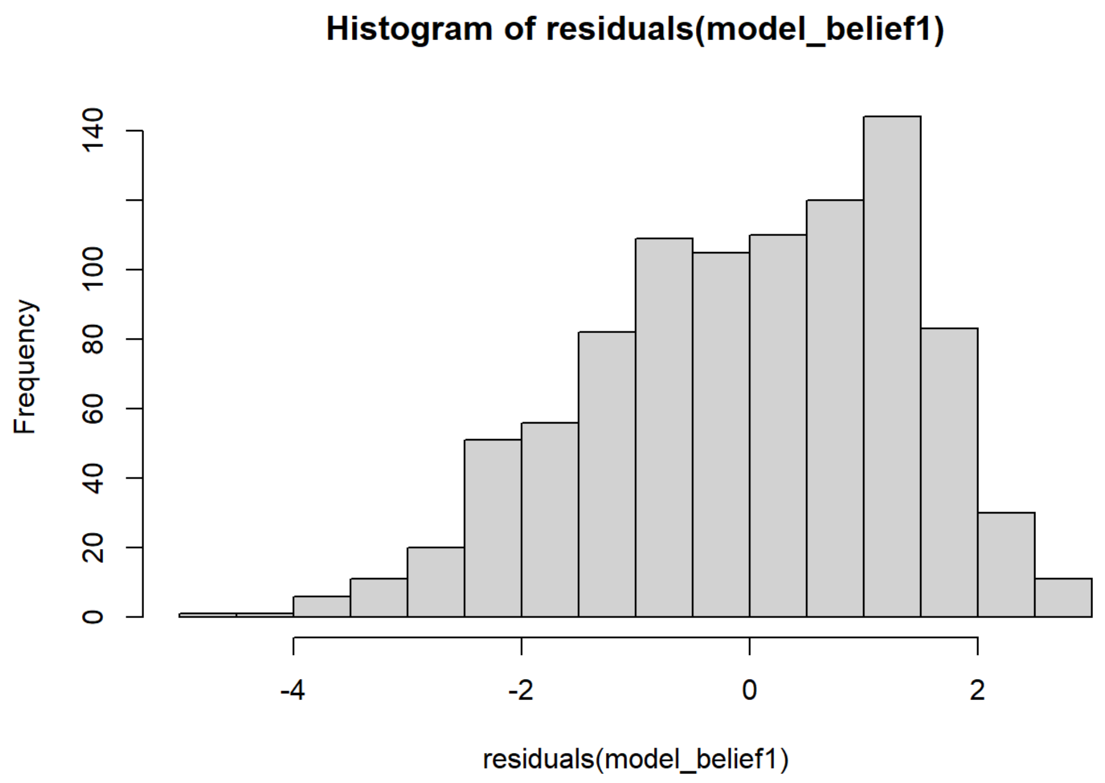

RQ1: Subscale Solve

```
model_solve0 <- lmer(pa_sol_solve ~ stage + (1| study_id), data = star)
model_solve1 <- lmer(pa_sol_solve ~ stage + (1 + stage | study_id), data = star)
```

```
## boundary (singular) fit: see help('isSingular')
```

```
anova(model_solve0, model_solve1)
```

```
## refitting model(s) with ML (instead of REML)
```

	npar <dbl>	AIC <dbl>	BIC <dbl>	logLik <dbl>	deviance <dbl>	Chisq <dbl>	Df <dbl>	Pr(>Chisq) <dbl>
model_solve0	4	3666.513	3685.909	-1829.256	3658.513	NA	NA	NA
model_solve1	6	3629.692	3658.787	-1808.846	3617.692	40.82073	2	1.367386e-09

2 rows

```
Anova(model_solve1)
```

	Chisq <dbl>	Df <dbl>	Pr(>Chisq) <dbl>
stage	79.32407	1	5.27135e-19

1 row

```
# Linearity
plot(resid(model_solve1), main="Testing linearity of the model")
```

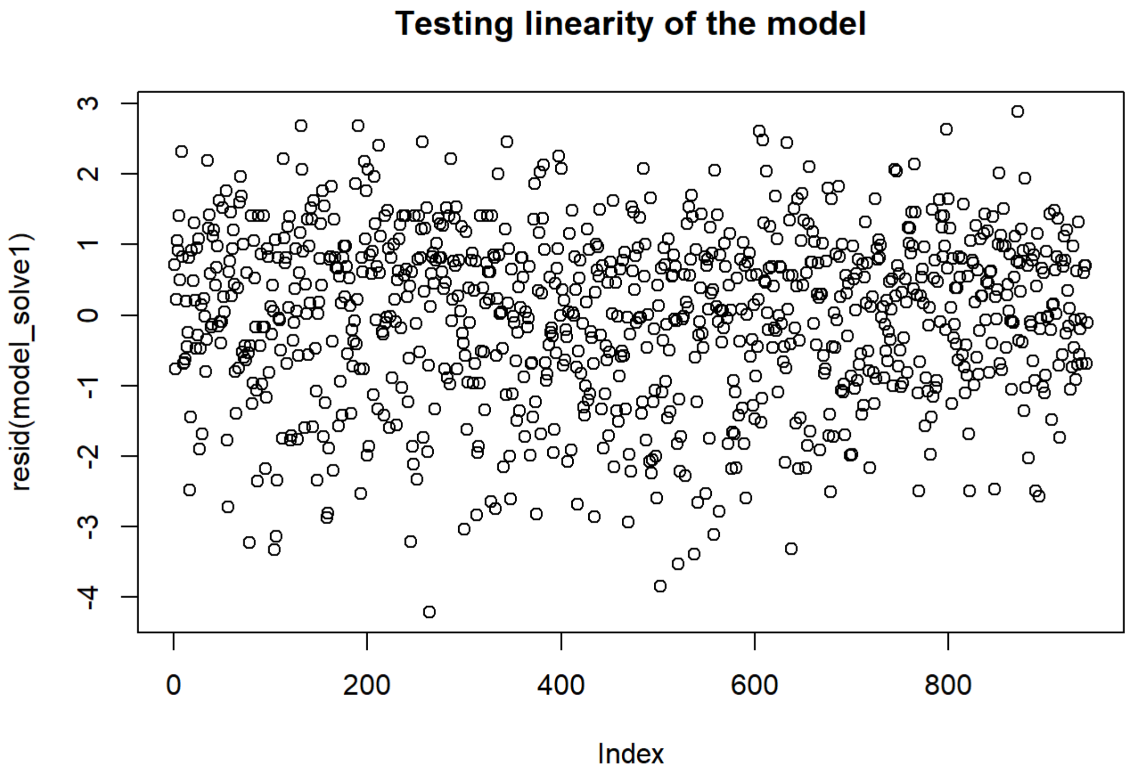


```
# Homogeneity of Variance: Levene Test
star1 <- star[!is.na(star$pa_sol_solve),]
star1$res2 <- abs(residuals(model_solve1))^2

anova(lm(star1$res2 ~ study_id, data = star1))
```

	Df <int>	Sum Sq <dbl>	Mean Sq <dbl>	F value <dbl>	Pr(>F) <dbl>
study_id	1	0.8909788	0.8909788	0.2235857	0.6364309
Residuals	941	3749.8424390	3.9849548	NA	NA

2 rows

```
# Normal distribution of residuals

qqmath(model_solve1, id = 0.05)
```

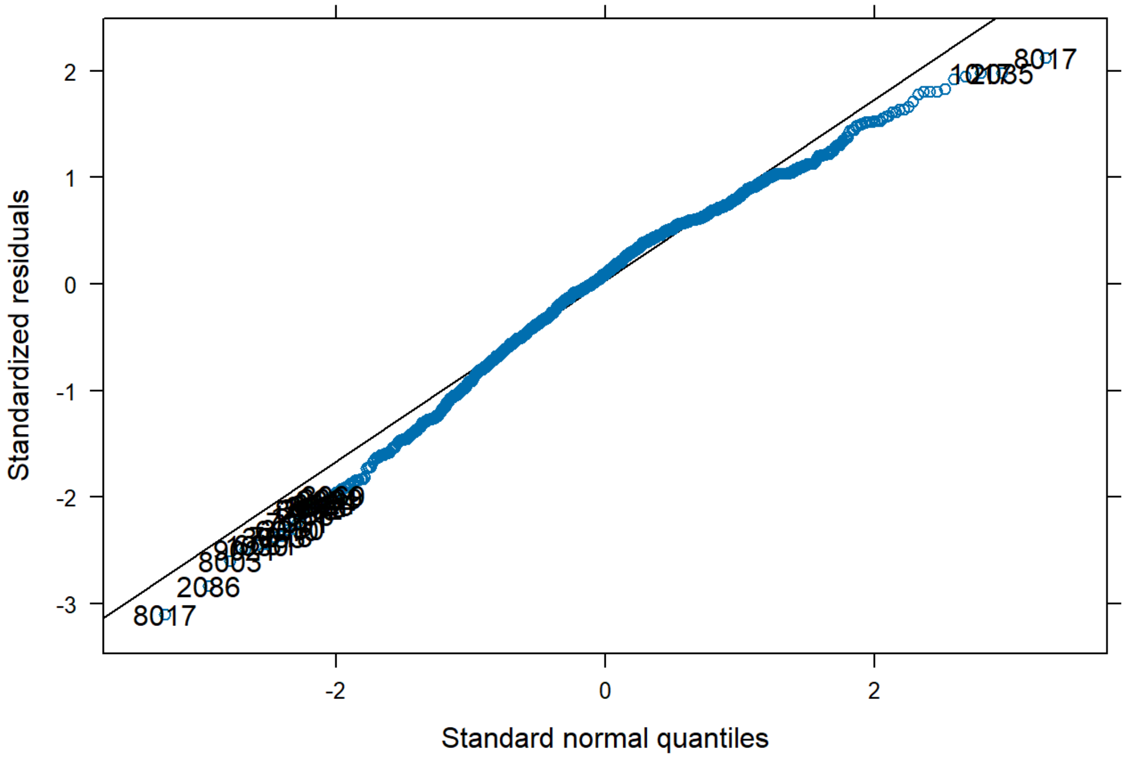


```
hist(residuals(model_solve1))
```

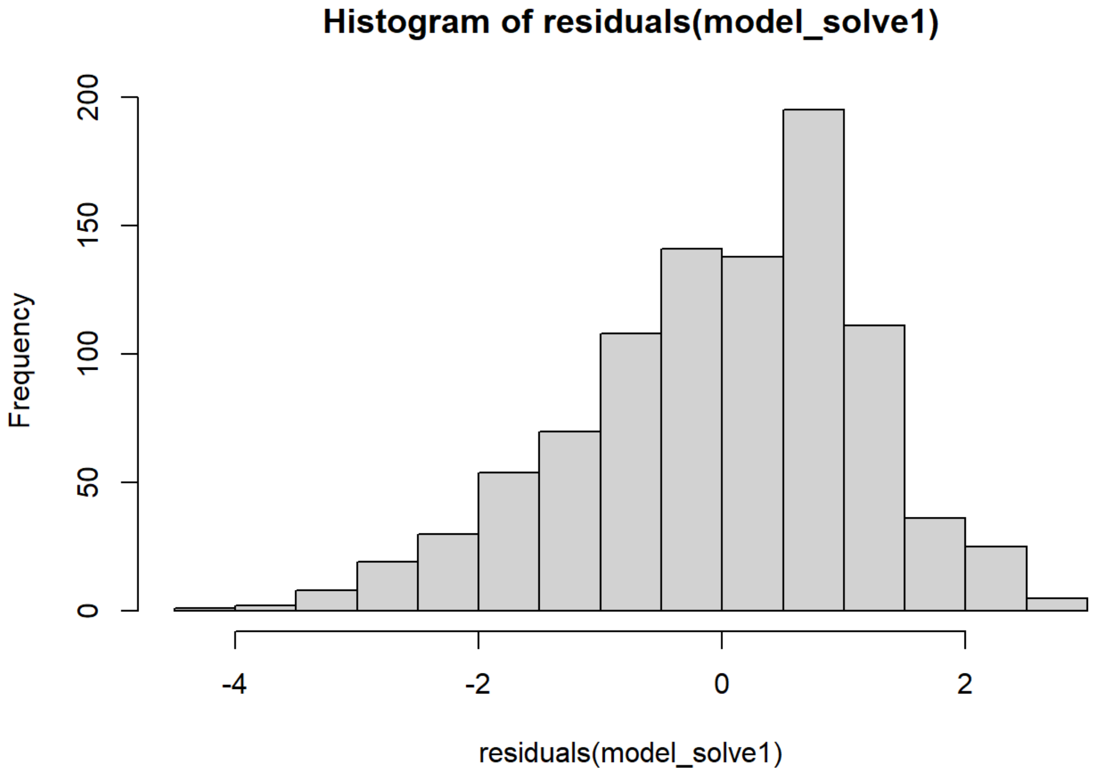

RQ1: Subscale Meaning

```
model_meaning0 <- lmer(pa_sol_meaning ~ stage + (1| study_id), data = star)
model_meaning1 <- lmer(pa_sol_meaning ~ stage + (1 + stage | study_id), data = star)
```

```
## boundary (singular) fit: see help('isSingular')
```

```
anova(model_meaning0, model_meaning1)
```

```
## refitting model(s) with ML (instead of REML)
```

	npar <dbl>	AIC <dbl>	BIC <dbl>	logLik <dbl>	deviance <dbl>	Chisq <dbl>	Df <dbl>	Pr(>Chisq) <dbl>
model_meaning0	4	3409.416	3428.821	-1700.708	3401.416	NA	NA	NA
model_meaning1	6	3352.850	3381.957	-1670.425	3340.850	60.56587	2	7.051629e-14

2 rows

```
Anova(model_meaning1)
```

	Chisq <dbl>	Df <dbl>	Pr(>Chisq) <dbl>
stage	19.97196	1	7.858632e-06

1 row

```
# Linearity
plot(resid(model_meaning1), main="Testing linearity of the model")
```

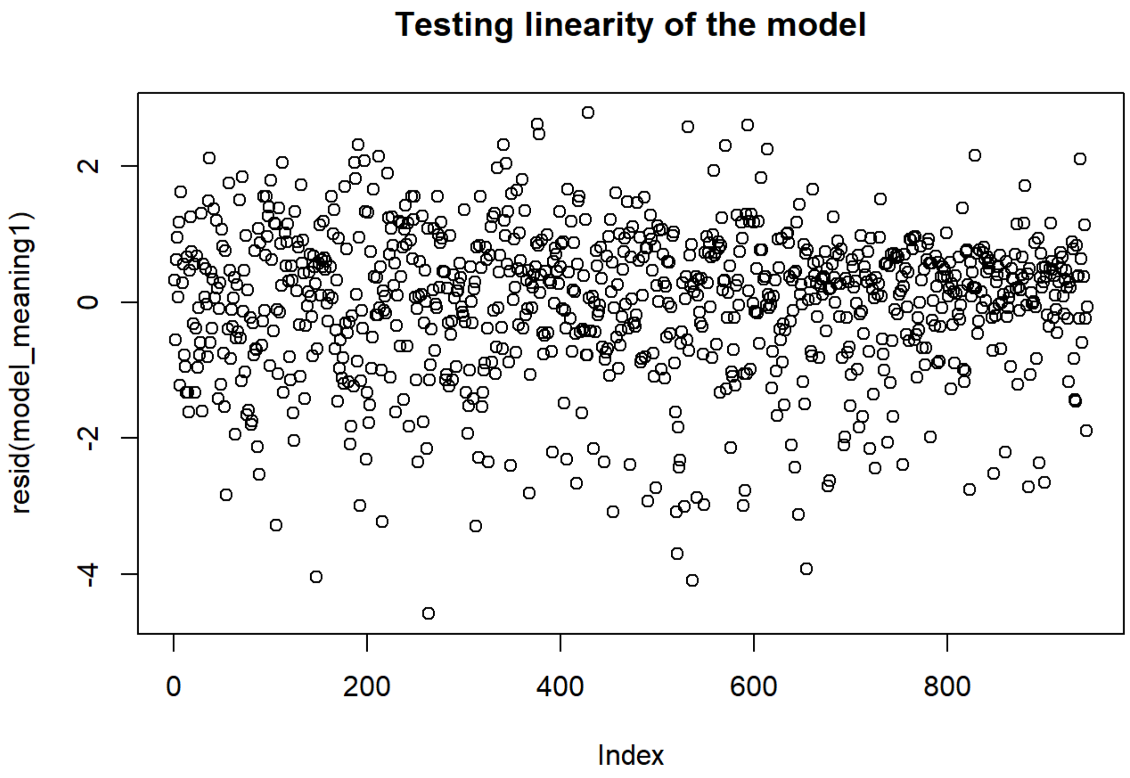


```
# Homogeneity of Variance: Levene Test
star1 <- star[!is.na(star$pa_sol_meaning),]
star1$res2 <- abs(residuals(model_meaning1))^2

anova(lm(star1$res2 ~ study_id, data = star1))
```

	Df <int>	Sum Sq <dbl>	Mean Sq <dbl>	F value <dbl>	Pr(>F) <dbl>
study_id	1	6.554684	6.554684	1.536683	0.2154206
Residuals	943	4022.343843	4.265476	NA	NA

2 rows

```
# Normal distribution of residuals

qqmath(model_meaning1, id = 0.05)
```

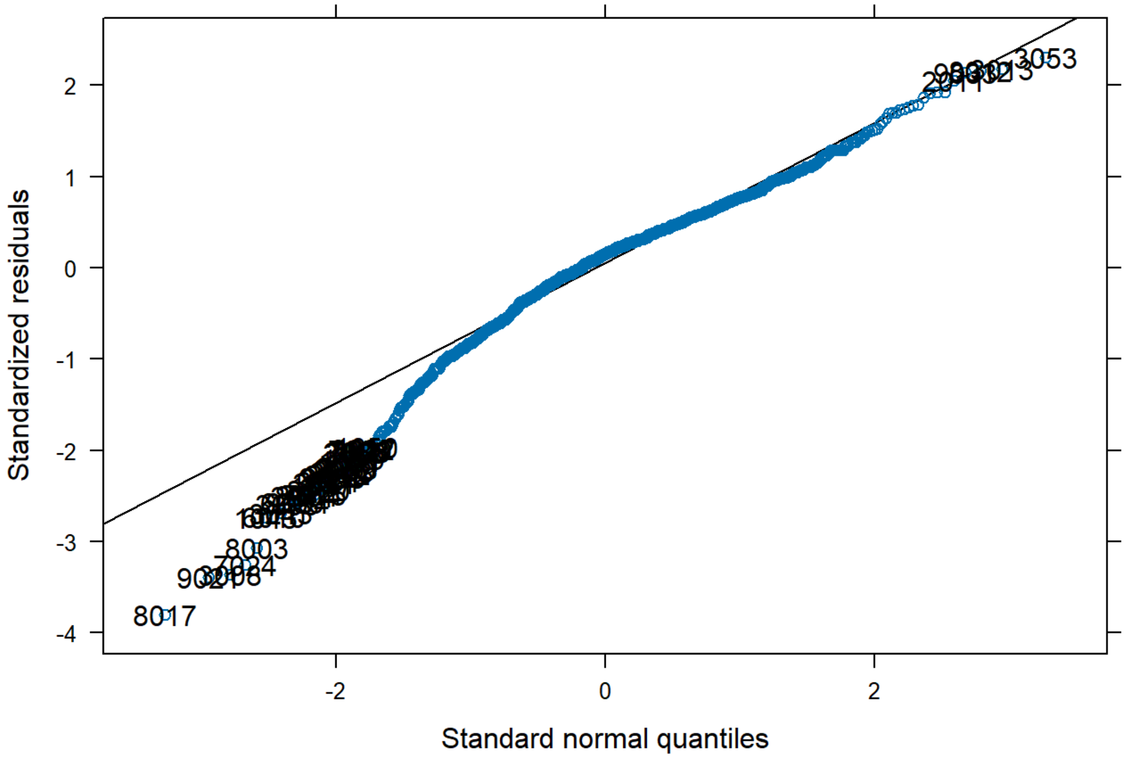


```
shapiro.test(residuals(model_meaning1))
```

```
##
##  Shapiro-Wilk normality test
##
## data:  residuals(model_meaning1)
## W = 0.95925, p-value = 1.462e-15
```

```
hist(residuals(model_meaning1))
```

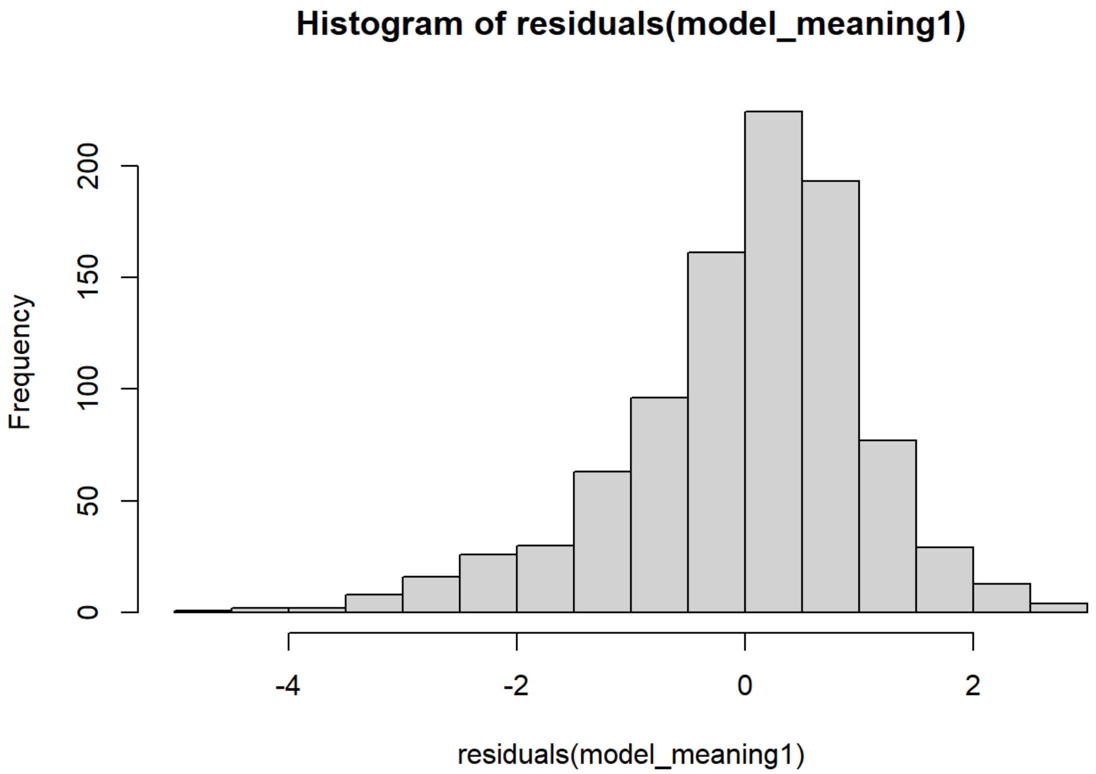

Which time points differ

Pairwise Comparisons for each Timepoint and each subscale

For each scale, we compare BL with FU1, BL with FU2, and FU1 with FU2. As can be seen in the table below, all comparisons are significant at an (uncorrected) 0.05 alpha level, with the exception of the sumscore and accept subscale between FU1 and FU2. Even if strictly correcting for multiple testing using Bonferroni (dividing alpha level by number of tests for each subscale, resulting in an alpha level of 0.0125), the majority of effects remain significant. It should be noted that mean differences are rather small!

```
timeline <- data.frame(
  Variable = character(),
  Comparison = character(),
  Mean_Difference = numeric(),
      t_statistic = numeric(),
  effectsize = numeric(),
  df = numeric(),
  p_value = numeric(),
  conf_lower = numeric(),
  conf_upper= numeric(),
  stringsAsFactors = FALSE
)

# Loop through variables and comparisons
for (variable in c("pa_sol", "pa_sol_accept", "pa_sol_meaning", "pa_sol_belief", "pa_sol_solv
e")) {
  for (comparison in c("BL-FU1", "BL-FU2", "FU1-FU2")) {
    # Extract relevant column names
    BL_col <- paste0(variable, "_BL")
    FU1_col <- paste0(variable, "_FU1")
    FU2_col <- paste0(variable, "_FU2")

    # Perform t-test
    if (comparison == "BL-FU1") {
      t_test_result <- t.test(star_wide[[FU1_col]], star_wide[[BL_col]], paired = TRUE)
      cohensD <- cohensD(star_wide[[BL_col]], star_wide[[FU1_col]], method = "paired")
    } else if (comparison == "BL-FU2") {
      t_test_result <- t.test(star_wide[[FU2_col]], star_wide[[BL_col]], paired = TRUE)
      cohensD <- cohensD(star_wide[[BL_col]], star_wide[[FU2_col]], method = "paired")
    } else if (comparison == "FU1-FU2") {
      t_test_result <- t.test(star_wide[[FU2_col]], star_wide[[FU1_col]], paired = TRUE)
      cohensD <- cohensD(star_wide[[FU1_col]], star_wide[[FU2_col]], method = "paired")
    }

    # Store results in the dataframe
    timeline <- rbind(timeline, data.frame(
      Variable = variable,
      Comparison = comparison,
      Mean_Difference = round(t_test_result$estimate,2),
      t_statistic = round(t_test_result$statistic,2),
      df = t_test_result$parameter,
      effectsize = round(cohensD,2),
      p_value = round(t_test_result$p.value,3),
      conf_lower = round(t_test_result$conf.int[1],3),
      conf_upper = round(t_test_result$conf.int[2],3),
      stringsAsFactors = FALSE
    ))
  }
}
rownames(timeline) <- NULL

kable(timeline)%>%
  kable_styling()
```

Variable	Comparison	Mean_Difference	t_statistic	df	effectsize	p_value	conf_lower	conf_upper
pa_sol	BL-FU1	-0.29	-5.35	291	0.31	0.000	-0.397	-0.183
pa_sol	BL-FU2	-0.27	-4.50	275	0.27	0.000	-0.395	-0.154
pa_sol	FU1-FU2	0.03	0.49	255	0.03	0.624	-0.084	0.139

Variable	Comparison	Mean_Difference	t_statistic	df	effectsize	p_value	conf_lower	conf_upper
pa_sol_accept	BL-FU1	0.38	3.16	291	0.18	0.002	0.143	0.615
pa_sol_accept	BL-FU2	0.31	2.44	275	0.15	0.015	0.060	0.565
pa_sol_accept	FU1-FU2	-0.13	-1.08	255	0.07	0.280	-0.371	0.108
pa_sol_meaning	BL-FU1	-0.31	-2.91	298	0.17	0.004	-0.527	-0.102
pa_sol_meaning	BL-FU2	-0.53	-4.66	282	0.28	0.000	-0.755	-0.307
pa_sol_meaning	FU1-FU2	-0.27	-2.42	261	0.15	0.016	-0.481	-0.050
pa_sol_belief	BL-FU1	-0.94	-7.25	294	0.42	0.000	-1.200	-0.688
pa_sol_belief	BL-FU2	-1.43	-9.87	280	0.59	0.000	-1.720	-1.148
pa_sol_belief	FU1-FU2	-0.58	-4.14	259	0.26	0.000	-0.854	-0.303
pa_sol_solve	BL-FU1	-0.78	-6.85	297	0.40	0.000	-1.009	-0.558
pa_sol_solve	BL-FU2	-1.05	-8.34	281	0.50	0.000	-1.298	-0.802
pa_sol_solve	FU1-FU2	-0.35	-2.89	260	0.18	0.004	-0.584	-0.111

```
write_xlsx(timeline, "timeline.xlsx")
```

Research Question 2: Does allocation to the STAR treatment pathway (vs care as usual) influence a change in pain problem solving style in adults who have received a total knee replacement due to osteoarthritis?

We use a random slope for stage based on the previous RQ

Accept

```
model_treatment_accept <- lmer(pa_sol_accept ~ stage*ou_arm + (1 + stage| study_id), data = sta
r)

anova(model_treatment_accept, model_accept1)
```

```
## refitting model(s) with ML (instead of REML)
```

	n...	AIC	BIC	logLik	deviance	Chisq	Df	Pr(>Chisq)
	<dbl>	<dbl>	<dbl>	<dbl>	<dbl>	<dbl>	<dbl>	<dbl>
model_accept1	6	3601.934	3630.958	-1794.967	3589.934	NA	NA	NA
model_treatment_accept	8	3605.606	3644.305	-1794.803	3589.606	0.3276126	2	0.8489064

2 rows

```
Anova(model_treatment_accept)
```

	Chisq <dbl>	Df <dbl>	Pr(>Chisq) <dbl>
stage	4.8061354	1	0.02835857
ou_arm	0.1491502	1	0.69934880
stage:ou_arm	0.1760724	1	0.67477055

3 rows

```
hist(residuals(model_treatment_accept))
```

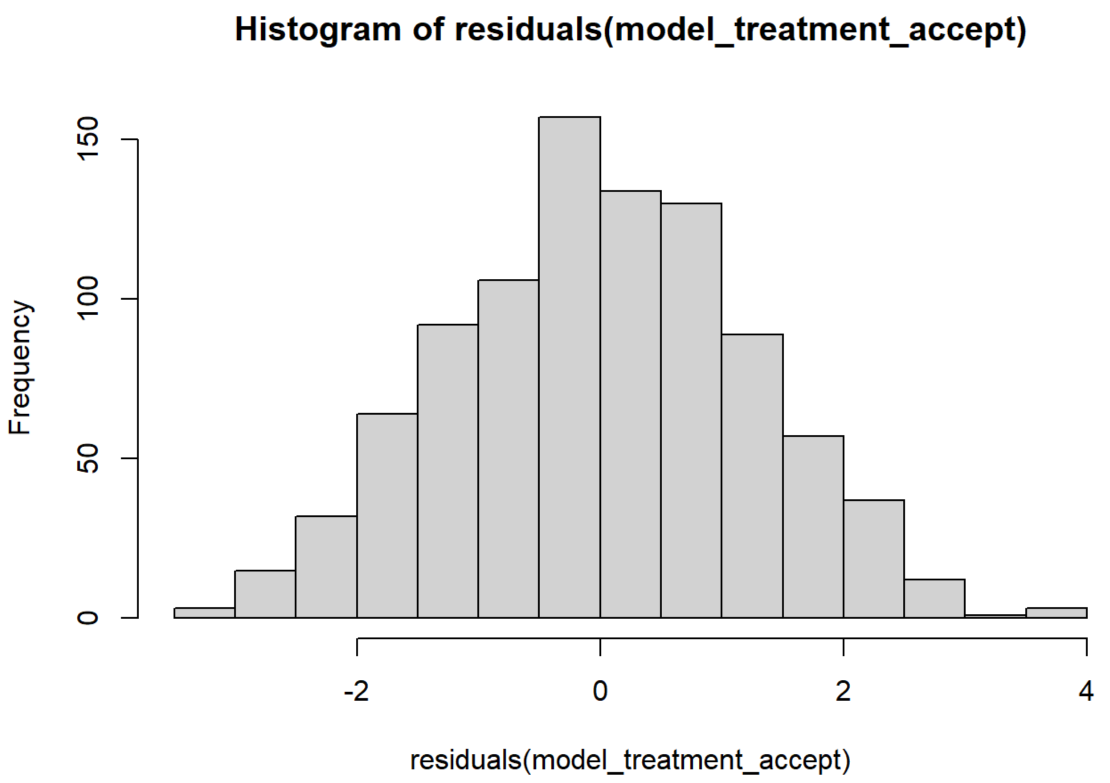

Meaning

```
model_treatment_meaning <- lmer(pa_sol_meaning ~ stage*ou_arm + (1 + stage | study_id), data = s
tar)
```

```
## boundary (singular) fit: see help('isSingular')
```

```
anova(model_treatment_meaning, model_meaning1)
```

```
## refitting model(s) with ML (instead of REML)
```

	n... <dbl>	AIC <dbl>	BIC <dbl>	logLik <dbl>	deviance <dbl>	Chisq <dbl>	Df <dbl>	Pr(>Chisq) <dbl>
model_meaning1	6	3352.850	3381.957	-1670.425	3340.850	NA	NA	NA
model_treatment_meaning	8	3356.431	3395.241	-1670.216	3340.431	0.4187288	2	0.8110996

2 rows

```
Anova(model_treatment_meaning)
```

	Chisq <dbl>	Df <dbl>	Pr(>Chisq) <dbl>
stage	19.93725767	1	8.002552e-06
ou_arm	0.09126693	1	7.625727e-01
stage:ou_arm	0.32570212	1	5.682009e-01

3 rows

```
hist(residuals(model_treatment_meaning))
```

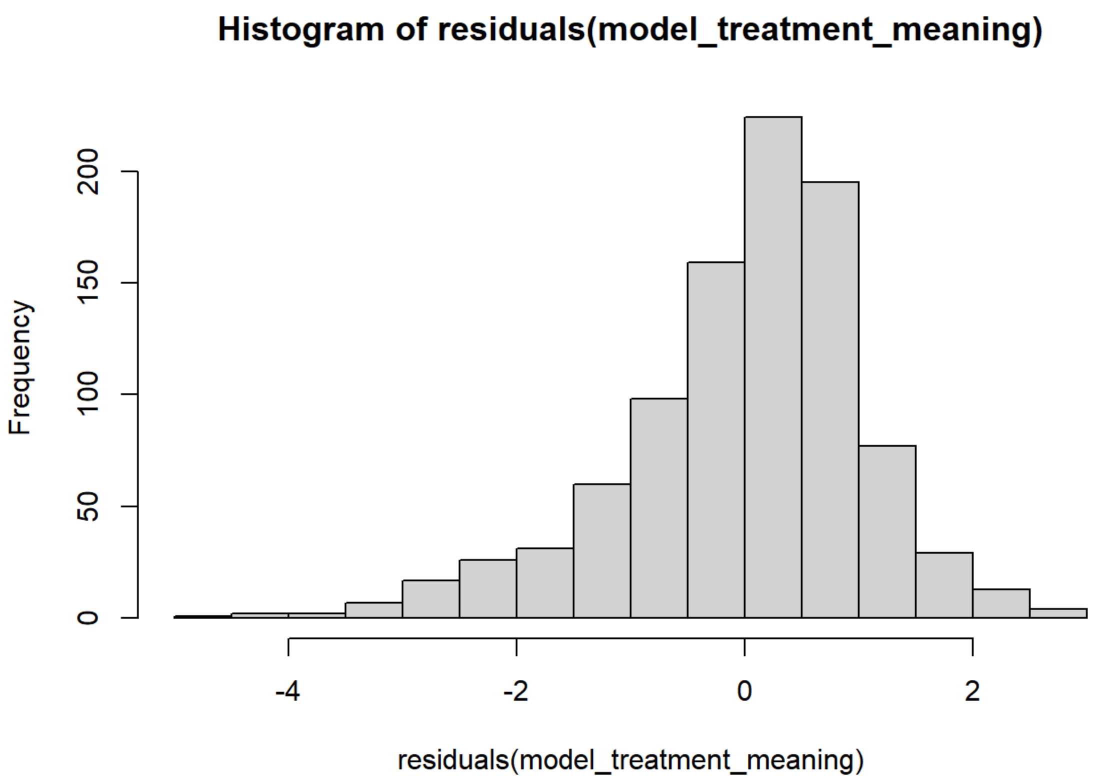

Solve

```
model_treatment_solve <- lmer(pa_sol_solve ~ stage*ou_arm + (1 + stage | study_id), data = star)
```

```
## boundary (singular) fit: see help('isSingular')
```

```
anova(model_treatment_solve, model_solve1)
```

```
## refitting model(s) with ML (instead of REML)
```

	n... <dbl>	AIC <dbl>	BIC <dbl>	logLik <dbl>	deviance <dbl>	Chisq <dbl>	Df <dbl>	Pr(>Chisq) <dbl>
model_solve1	6	3629.692	3658.787	-1808.846	3617.692	NA	NA	NA
model_treatment_solve	8	3632.469	3671.261	-1808.234	3616.469	1.223322	2	0.5424491

2 rows

```
Anova(model_treatment_solve)
```

	Chisq<br><dbl>	Df<br><dbl>	Pr(>Chisq)<br><dbl>
stage	79.3836656	1	5.114723e-19
ou_arm	0.1495106	1	6.990035e-01
stage:ou_arm	1.0710121	1	3.007169e-01

3 rows

```
hist(residuals(model_treatment_solve))
```

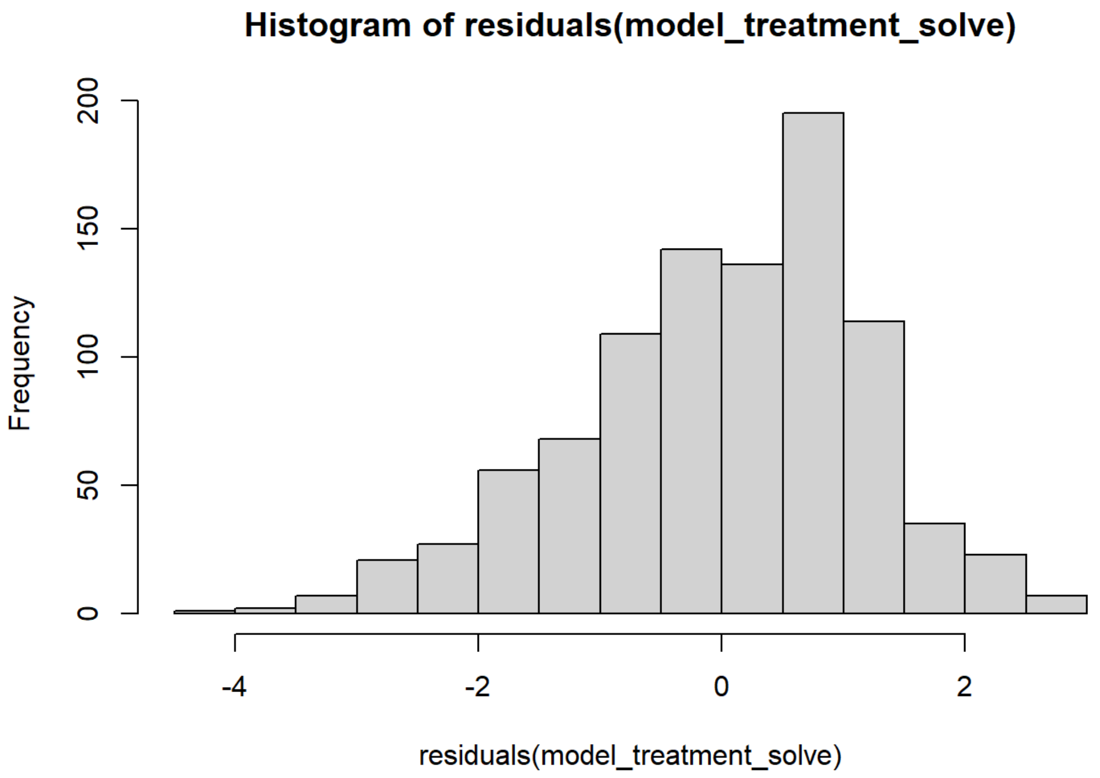

Belief

```
model_treatment_belief <- lmer(pa_sol_belief ~ stage*ou_arm + (1 + stage| study_id), data = sta
r)
```

```
## boundary (singular) fit: see help('isSingular')
```

```
anova(model_treatment_belief, model_belief1)
```

```
## refitting model(s) with ML (instead of REML)
```

	n...<br><dbl>	AIC<br><dbl>	BIC<br><dbl>	logLik<br><dbl>	deviance<br><dbl>	Chisq<br><dbl>	Df<br><dbl>	Pr(>Chisq)<br><dbl>
model_belief1	6	3771.701	3800.776	-1879.851	3759.701	NA	NA	NA
model_treatment_belief	8	3775.401	3814.169	-1879.701	3759.401	0.2995658	2	0.8608949

2 rows

```
Anova(model_treatment_belief)
```

	Chisq <dbl>	Df <dbl>	Pr(>Chisq) <dbl>
stage	104.69804516	1	1.422413e-24
ou_arm	0.05074952	1	8.217642e-01
stage:ou_arm	0.24784296	1	6.185980e-01

3 rows

```
hist(residuals(model_treatment_belief))
```

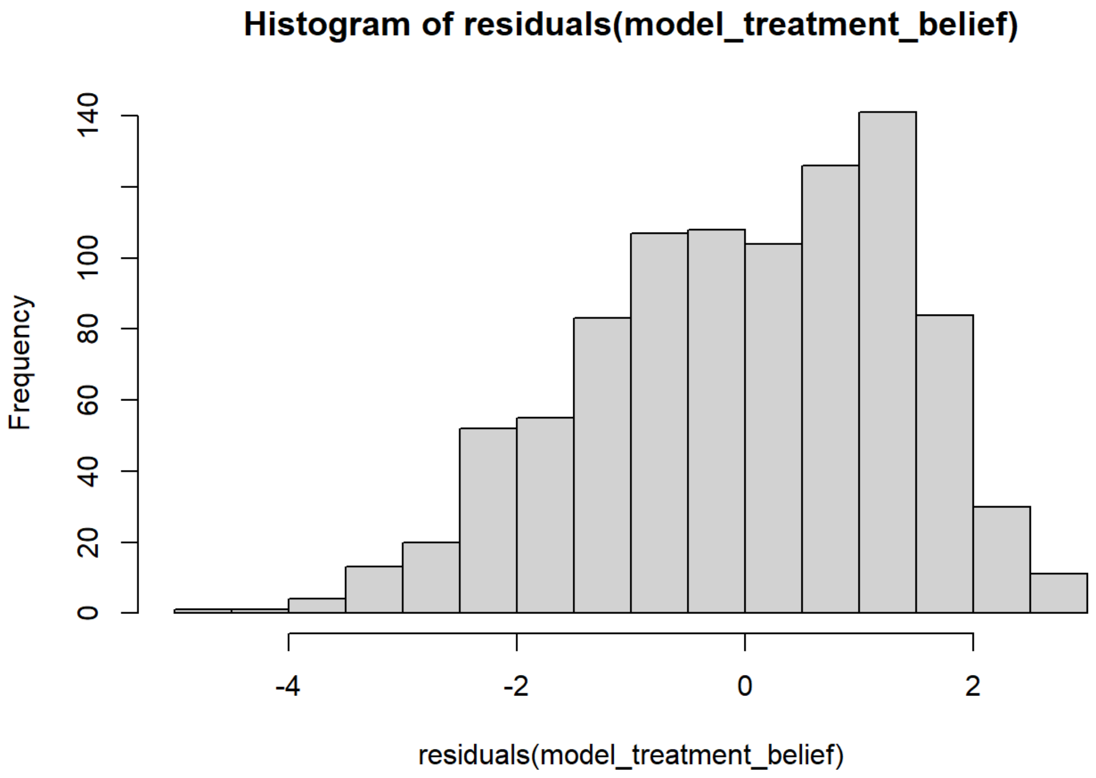

Research Question 3: Does the pain problem solving style a patient adopts 3 months post-total knee replacement predict pain severity and interference 15 months post-operation? Is this moderated by treatment pathway allocation?

This is not a multilevel problem.

We run a linear regression model predicting pain severity, interference at 15 months using the interaction of treatment path way and pa_sol.

Pain Severity

```
model_sev_subscales <- lm(bpi_severity_FU2 ~ bpi_severity_BL +
                          pa_sol_accept_BL  +
                          pa_sol_belief_BL +
                          pa_sol_meaning_BL+
                          pa_sol_solve_BL , data = star_wide)


round(Anova(model_sev_subscales),2)
```

	Sum Sq <dbl>	Df <dbl>	F value <dbl>	Pr(>F) <dbl>
bpi_severity_BL	294.89	1	61.91	0.00
pa_sol_accept_BL	5.67	1	1.19	0.28
pa_sol_belief_BL	0.64	1	0.13	0.71
pa_sol_meaning_BL	23.63	1	4.96	0.03
pa_sol_solve_BL	0.66	1	0.14	0.71
Residuals	1433.84	301	NA	NA

6 rows

```
summary(model_sev_subscales)
```

```
##
## Call:
## lm(formula = bpi_severity_FU2 ~ bpi_severity_BL + pa_sol_accept_BL +
##     pa_sol_belief_BL + pa_sol_meaning_BL + pa_sol_solve_BL, data = star_wide)
##
## Residuals:
##     Min      1Q  Median      3Q     Max
## -4.9428 -1.4445 -0.3074  1.4750  7.9813
##
## Coefficients:
##                   Estimate Std. Error t value Pr(>|t|)
## (Intercept)        1.32744    0.76130   1.744   0.0822 .
## bpi_severity_BL    0.64129    0.08151   7.868 6.53e-14 ***
## pa_sol_accept_BL   0.09161    0.08400   1.091   0.2763
## pa_sol_belief_BL  -0.03621    0.09898  -0.366   0.7147
## pa_sol_meaning_BL -0.29984    0.13461  -2.227   0.0267 *
## pa_sol_solve_BL    0.03481    0.09317   0.374   0.7089
## ---
## Signif. codes:  0 '***' 0.001 '**' 0.01 '*' 0.05 '.' 0.1 ' ' 1
##
## Residual standard error: 2.183 on 301 degrees of freedom
##   (56 observations deleted due to missingness)
## Multiple R-squared:  0.2344, Adjusted R-squared:  0.2217
## F-statistic: 18.43 on 5 and 301 DF,  p-value: 5.767e-16
```

```
hist(residuals(model_sev_subscales))
```

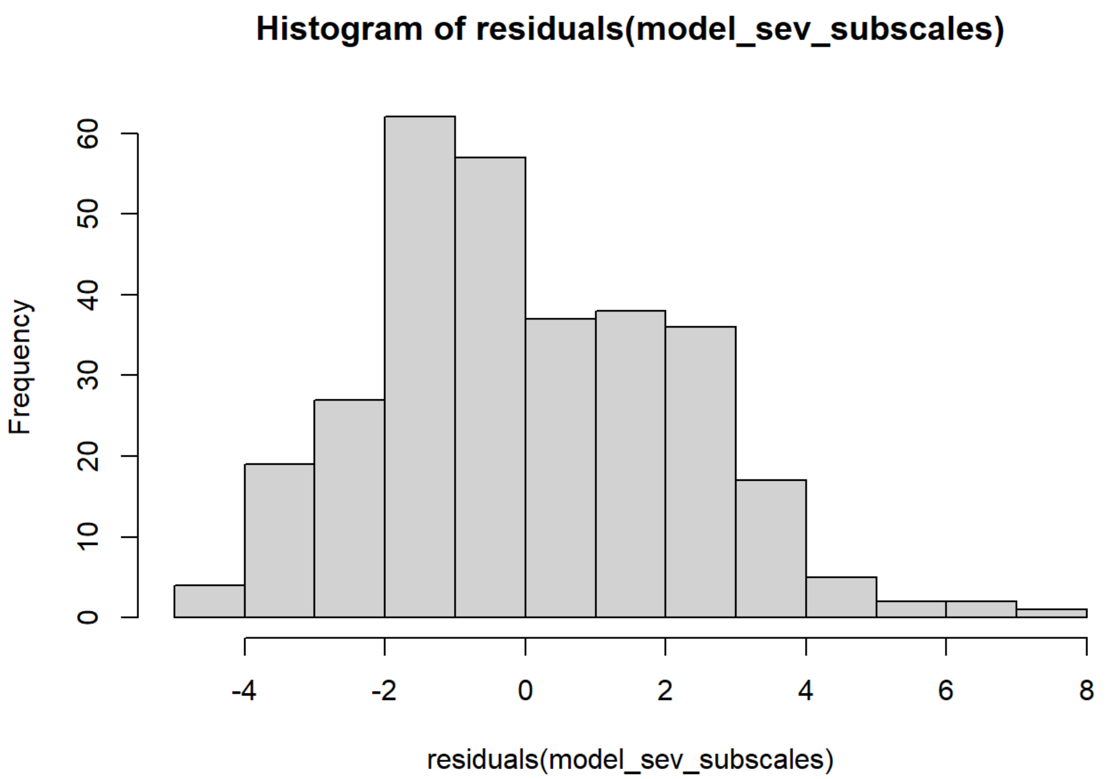


```
#ggplot(star_wide, aes(pa_sol_BL, bpi_severity_FU2)) +
#      geom_point(size = 2, aes(color= as.factor(ou_arm))) +
#      geom_smooth(aes(group = ou_arm, color = as.factor(ou_arm)), method = "lm", fill = "whit
e")

#ggplot(star_wide, aes(pa_sol_BL, bpi_severity_FU2)) +
#        geom_smooth(aes(), method = "lm", fill = "white") +
#        geom_point(size = 2, aes())


ggplot(star_wide, aes(pa_sol_meaning_BL, bpi_severity_FU2)) +
      geom_point(size = 2, aes(color= as.factor(ou_arm))) +
      geom_smooth(aes(group = ou_arm, color = as.factor(ou_arm)), method = "lm", fill = "white")
```

```
## `geom_smooth()` using formula = 'y ~ x'
```

```
## Warning: Removed 51 rows containing non-finite values (`stat_smooth()`).
```

```
## Warning: Removed 51 rows containing missing values (`geom_point()`).
```

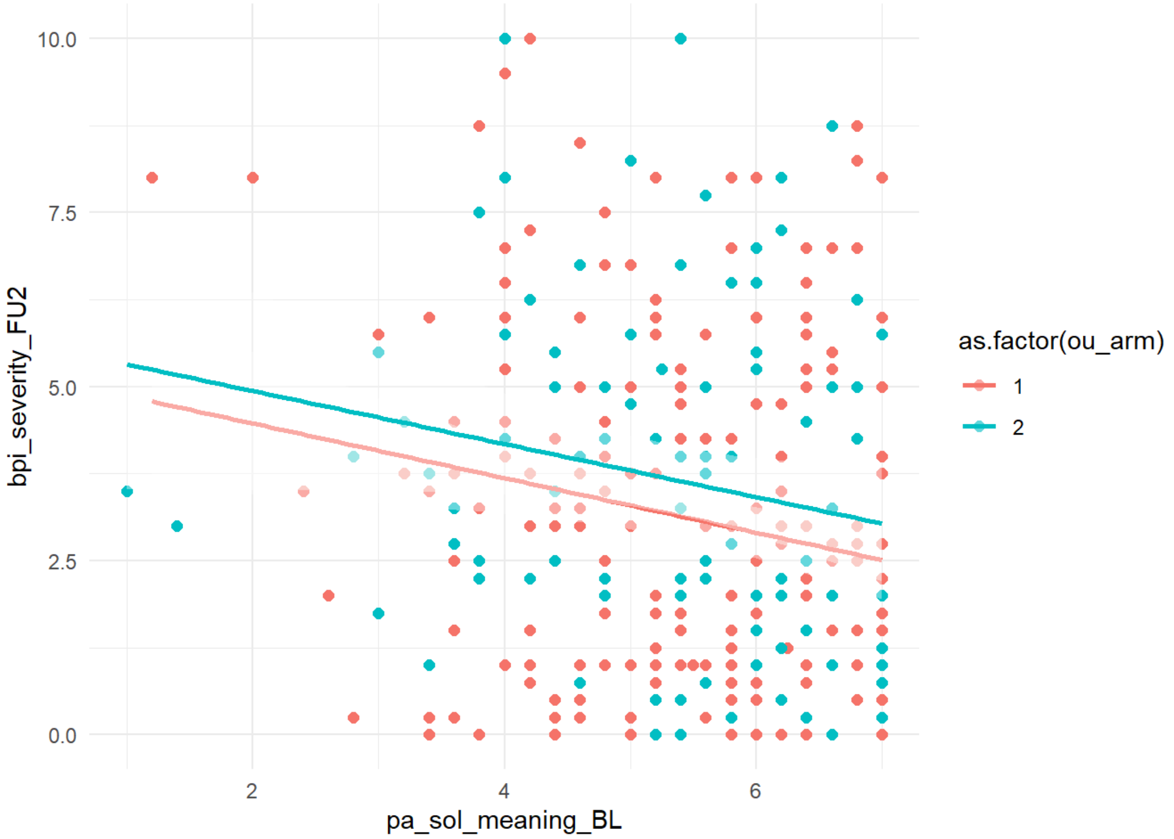


```
ggplot(star_wide, aes(pa_sol_meaning_BL, bpi_severity_FU2)) +
       geom_smooth(aes(), method = "lm", fill = "white") +
       geom_point(size = 2, aes())
```

```
## `geom_smooth()` using formula = 'y ~ x'
```

```
## Warning: Removed 51 rows containing non-finite values (`stat_smooth()`).
## Removed 51 rows containing missing values (`geom_point()`).
```

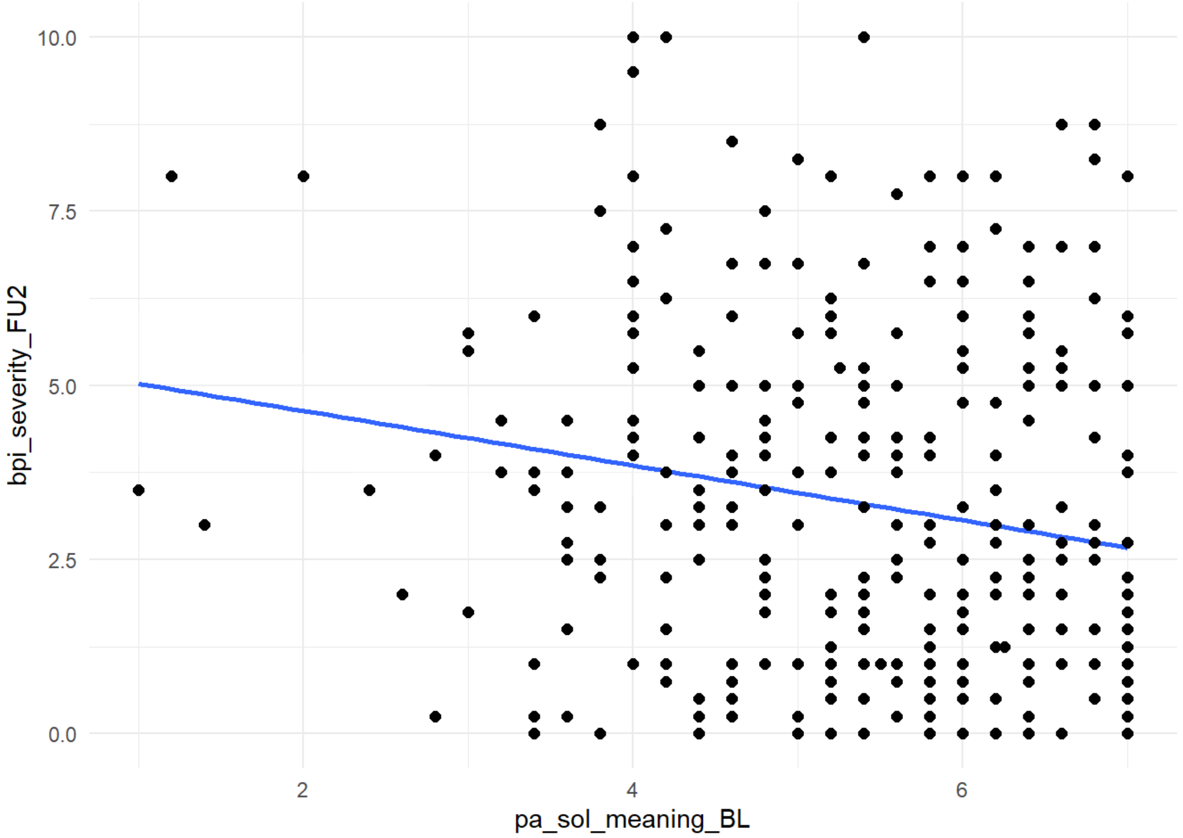

```
model_sev_subscales_mod <- lm(bpi_severity_FU2 ~ bpi_severity_BL +
                          pa_sol_accept_BL * ou_arm+
                          pa_sol_belief_BL * ou_arm+
                          pa_sol_meaning_BL* ou_arm +
                          pa_sol_solve_BL* ou_arm, data = star_wide)


round(Anova(model_sev_subscales_mod),2)
```

	Sum Sq <dbl>	Df <dbl>	F value <dbl>	Pr(>F) <dbl>
bpi_severity_BL	302.04	1	64.24	0.00
pa_sol_accept_BL	7.52	1	1.60	0.21
ou_arm	30.22	1	6.43	0.01
pa_sol_belief_BL	0.32	1	0.07	0.80
pa_sol_meaning_BL	25.64	1	5.45	0.02
pa_sol_solve_BL	1.20	1	0.26	0.61
pa_sol_accept_BL:ou_arm	7.32	1	1.56	0.21
ou_arm:pa_sol_belief_BL	3.86	1	0.82	0.37
ou_arm:pa_sol_meaning_BL	1.46	1	0.31	0.58
ou_arm:pa_sol_solve_BL	0.75	1	0.16	0.69

1-10 of 11 rows Previous **1** 2 Next

```
summary(model_sev_subscales_mod)
```

```
##
## Call:
## lm(formula = bpi_severity_FU2 ~ bpi_severity_BL + pa_sol_accept_BL *
##     ou_arm + pa_sol_belief_BL * ou_arm + pa_sol_meaning_BL *
##     ou_arm + pa_sol_solve_BL * ou_arm, data = star_wide)
##
## Residuals:
##     Min      1Q  Median      3Q     Max
## -4.7163 -1.4939 -0.3642  1.5152  7.9040
##
## Coefficients:
##                          Estimate Std. Error t value Pr(>|t|)
## (Intercept)               2.07063    2.05764   1.006    0.315
## bpi_severity_BL           0.65096    0.08122   8.015 2.57e-14 ***
## pa_sol_accept_BL         -0.18285    0.24611  -0.743    0.458
## ou_arm                   -0.64083    1.40771  -0.455    0.649
## pa_sol_belief_BL         -0.27646    0.29401  -0.940    0.348
## pa_sol_meaning_BL        -0.10836    0.39076  -0.277    0.782
## pa_sol_solve_BL          -0.05627    0.27390  -0.205    0.837
## pa_sol_accept_BL:ou_arm   0.21757    0.17441   1.247    0.213
## ou_arm:pa_sol_belief_BL   0.19054    0.21041   0.906    0.366
## ou_arm:pa_sol_meaning_BL -0.15450    0.27748  -0.557    0.578
## ou_arm:pa_sol_solve_BL    0.07344    0.18339   0.400    0.689
## ---
## Signif. codes:  0 '***' 0.001 '**' 0.01 '*' 0.05 '.' 0.1 ' ' 1
##
## Residual standard error: 2.168 on 296 degrees of freedom
##   (56 observations deleted due to missingness)
## Multiple R-squared:  0.2569, Adjusted R-squared:  0.2318
## F-statistic: 10.23 on 10 and 296 DF,  p-value: 8.302e-15
```

Pain Interference

```
model_subscales_int <- lm(bpi_int_FU2 ~  bpi_int_BL +
                          pa_sol_accept_BL+
                          pa_sol_belief_BL+
                          pa_sol_meaning_BL+
                          pa_sol_solve_BL , data = star_wide)

round(Anova(model_subscales_int),2)
```

	Sum Sq <dbl>	Df <dbl>	F value <dbl>	Pr(>F) <dbl>
bpi_int_BL	290.51	1	45.78	0.00
pa_sol_accept_BL	31.69	1	4.99	0.03
pa_sol_belief_BL	0.95	1	0.15	0.70
pa_sol_meaning_BL	49.29	1	7.77	0.01
pa_sol_solve_BL	18.28	1	2.88	0.09
Residuals	1916.32	302	NA	NA

6 rows

```
summary(model_subscales_int)
```

```
##
## Call:
## lm(formula = bpi_int_FU2 ~ bpi_int_BL + pa_sol_accept_BL + pa_sol_belief_BL +
##     pa_sol_meaning_BL + pa_sol_solve_BL, data = star_wide)
##
## Residuals:
##    Min     1Q Median     3Q    Max
## -5.665 -1.770 -0.292  1.912  6.812
##
## Coefficients:
##                   Estimate Std. Error t value Pr(>|t|)
## (Intercept)        1.05305    0.90311   1.166  0.24452
## bpi_int_BL         0.55245    0.08165   6.766 6.86e-11 ***
## pa_sol_accept_BL   0.21127    0.09454   2.235  0.02617 *
## pa_sol_belief_BL  -0.04368    0.11281  -0.387  0.69886
## pa_sol_meaning_BL -0.42447    0.15229  -2.787  0.00565 **
## pa_sol_solve_BL    0.18760    0.11051   1.698  0.09063 .
## ---
## Signif. codes:  0 '***' 0.001 '**' 0.01 '*' 0.05 '.' 0.1 ' ' 1
##
## Residual standard error: 2.519 on 302 degrees of freedom
##   (55 observations deleted due to missingness)
## Multiple R-squared:  0.2207, Adjusted R-squared:  0.2078
## F-statistic: 17.11 on 5 and 302 DF,  p-value: 6.701e-15
```

```
hist(residuals(model_subscales_int))
```

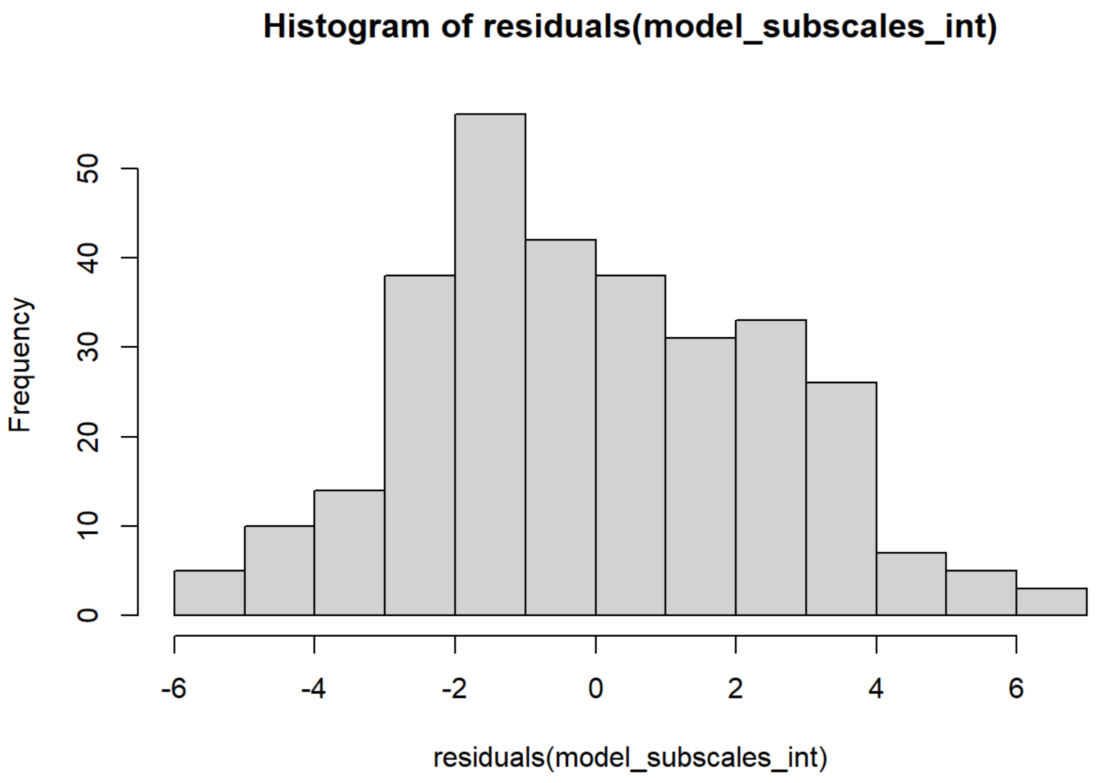

```
#ggplot(star_wide, aes(pa_sol_BL, bpi_int_FU2)) +
#      geom_point(size = 2, aes(color= as.factor(ou_arm))) +
#      geom_smooth(aes(group = ou_arm, color = as.factor(ou_arm)), method = "lm", fill = "whit
e")

#ggplot(star_wide, aes(pa_sol_BL, bpi_int_FU2)) +
#      geom_point(size = 2, aes()) +
#      geom_smooth(aes(), method = "lm", fill = "white")


ggplot(star_wide, aes(pa_sol_accept_BL, bpi_int_FU2)) +
      geom_point(size = 2, aes(color= as.factor(ou_arm))) +
      geom_smooth(aes(group = ou_arm, color = as.factor(ou_arm)), method = "lm", fill = "white")
```

```
## `geom_smooth()` using formula = 'y ~ x'
```

```
## Warning: Removed 52 rows containing non-finite values (`stat_smooth()`).
```

```
## Warning: Removed 52 rows containing missing values (`geom_point()`).
```

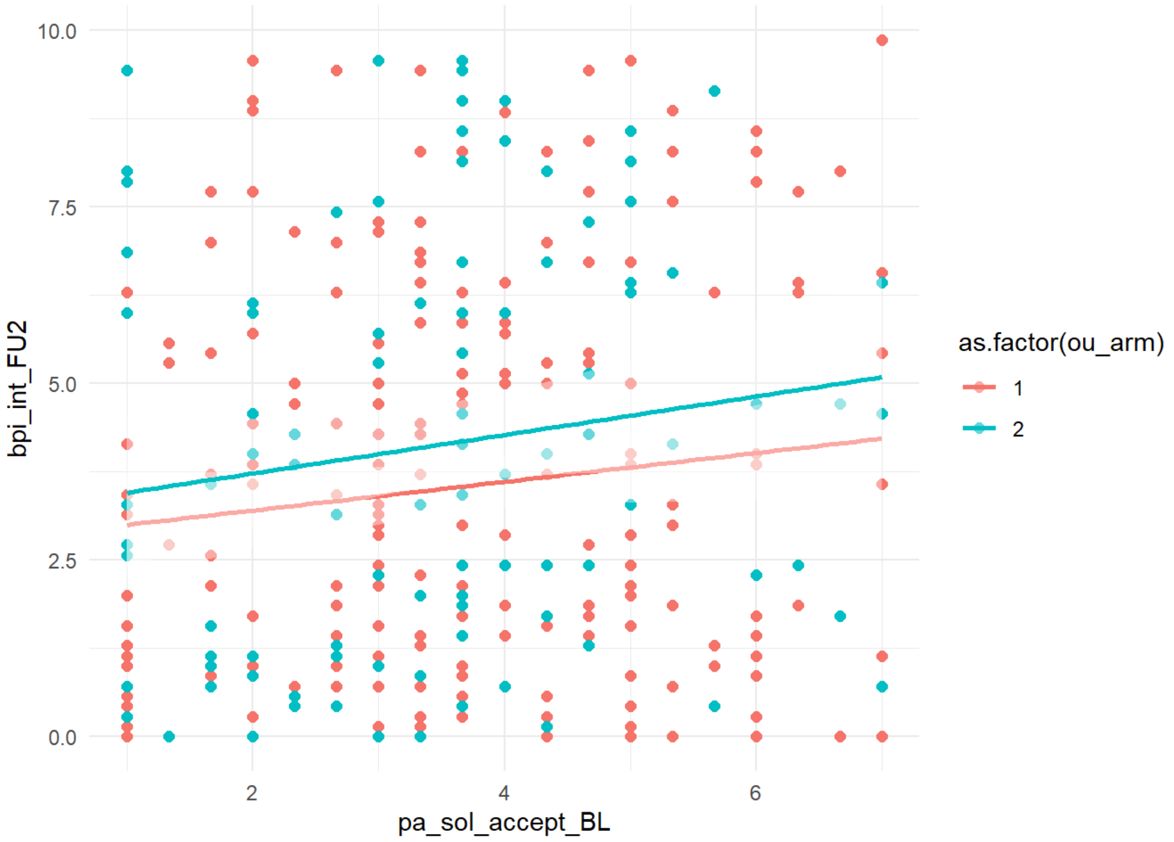


```
ggplot(star_wide, aes(pa_sol_accept_BL, bpi_int_FU2)) +
      geom_point(size = 2, aes()) +
      geom_smooth(aes(), method = "lm", fill = "white")
```

```
## `geom_smooth()` using formula = 'y ~ x'
```

```
## Warning: Removed 52 rows containing non-finite values (`stat_smooth()`).
## Removed 52 rows containing missing values (`geom_point()`).
```

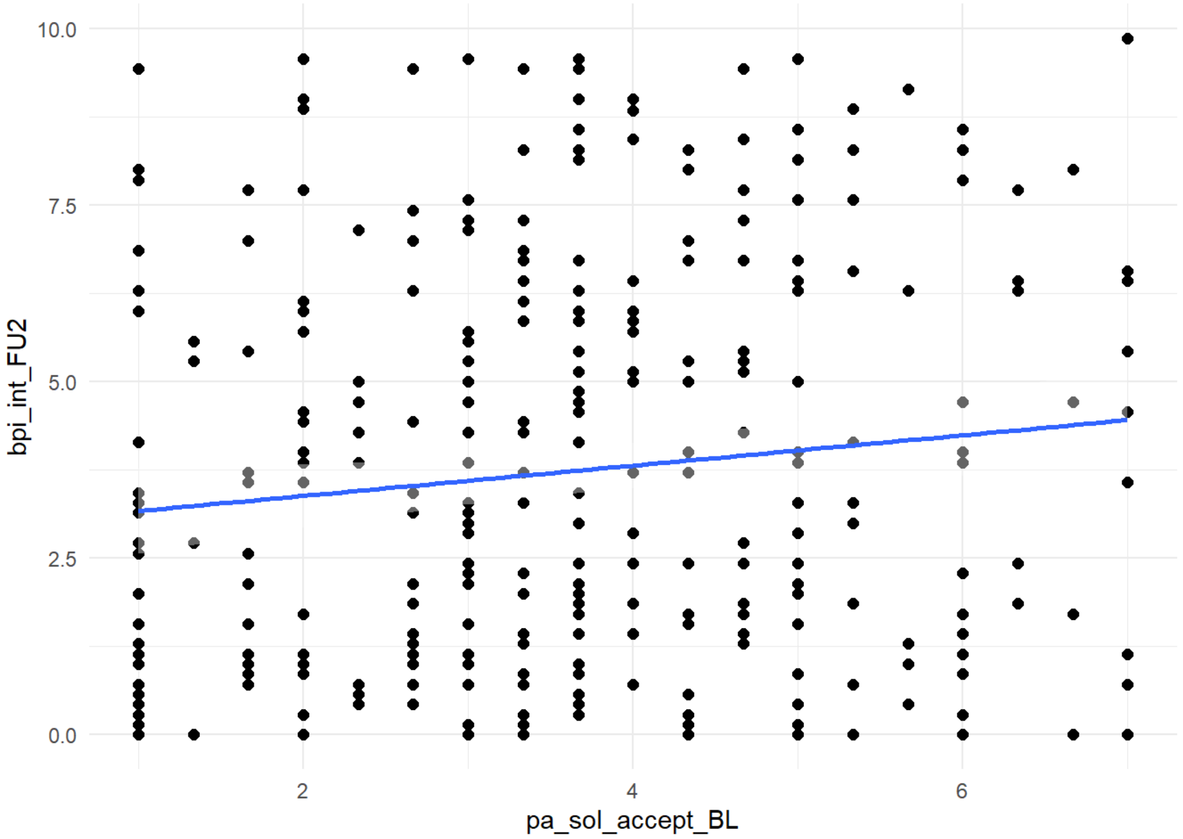


```
ggplot(star_wide, aes(pa_sol_meaning_BL, bpi_int_FU2)) +
      geom_point(size = 2, aes(color= as.factor(ou_arm))) +
      geom_smooth(aes(group = ou_arm, color = as.factor(ou_arm)), method = "lm", fill = "white")
```

```
## `geom_smooth()` using formula = 'y ~ x'
```

```
## Warning: Removed 50 rows containing non-finite values (`stat_smooth()`).
```

```
## Warning: Removed 50 rows containing missing values (`geom_point()`).
```

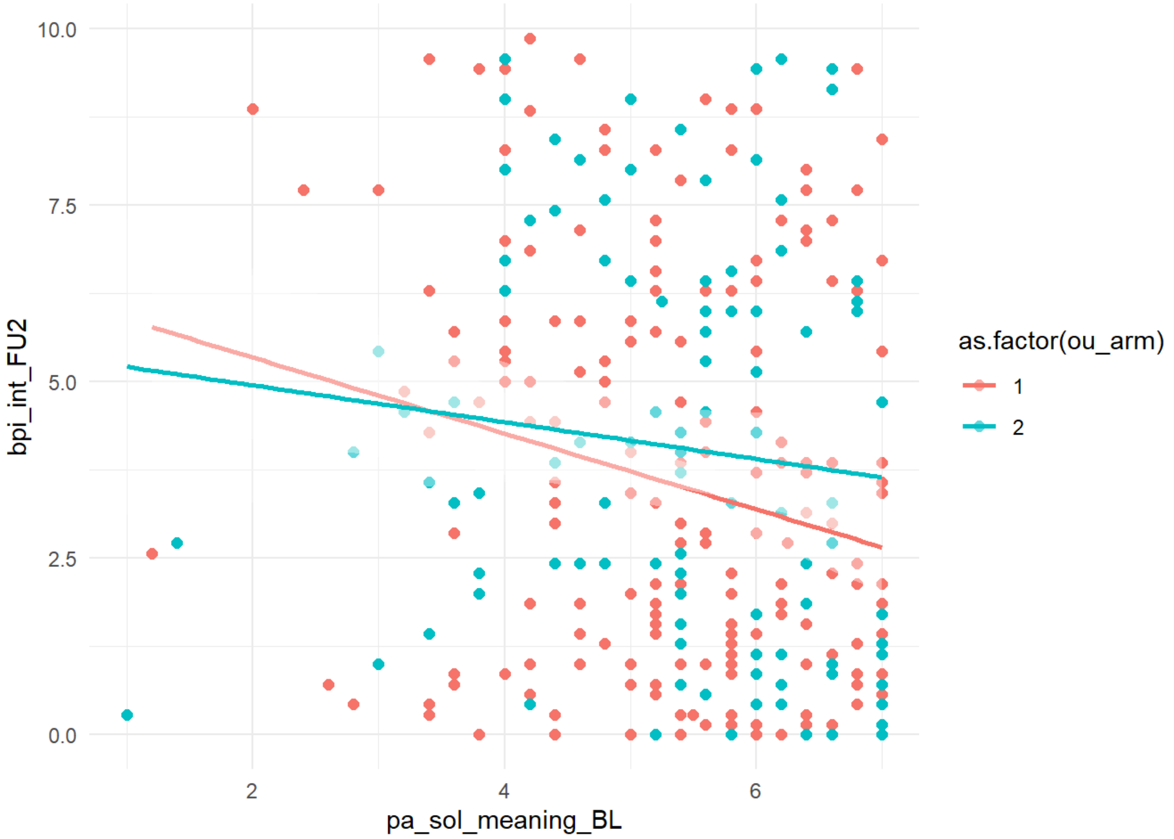


```
ggplot(star_wide, aes(pa_sol_meaning_BL, bpi_int_FU2)) +
      geom_point(size = 2, aes()) +
      geom_smooth(aes(), method = "lm", fill = "white")
```

```
## `geom_smooth()` using formula = 'y ~ x'
```

```
## Warning: Removed 50 rows containing non-finite values (`stat_smooth()`).
## Removed 50 rows containing missing values (`geom_point()`).
```

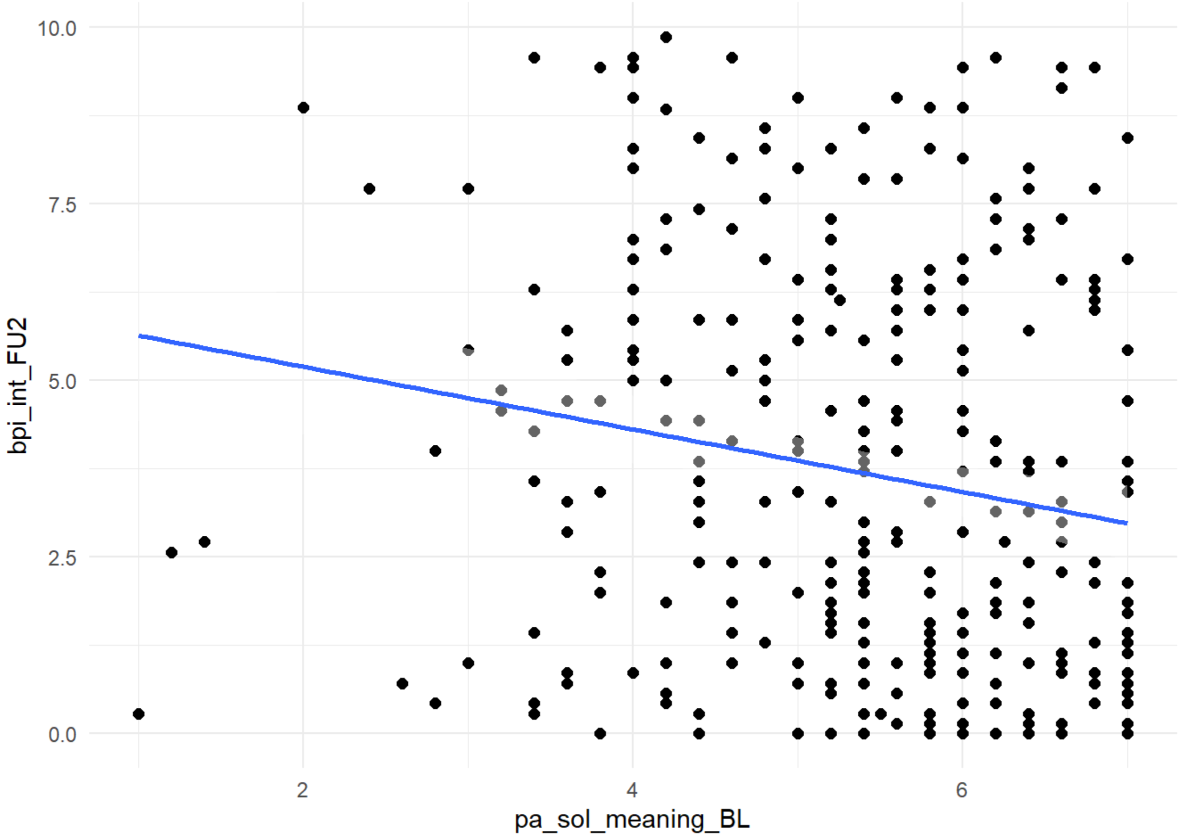

```
ggplot(star_wide, aes(pa_sol_solve_BL, bpi_int_FU2)) +
      geom_point(size = 2, aes(color= as.factor(ou_arm))) +
      geom_smooth(aes(group = ou_arm, color = as.factor(ou_arm)), method = "lm", fill = "white")
```

```
## `geom_smooth()` using formula = 'y ~ x'
```

```
## Warning: Removed 50 rows containing non-finite values (`stat_smooth()`).
## Removed 50 rows containing missing values (`geom_point()`).
```

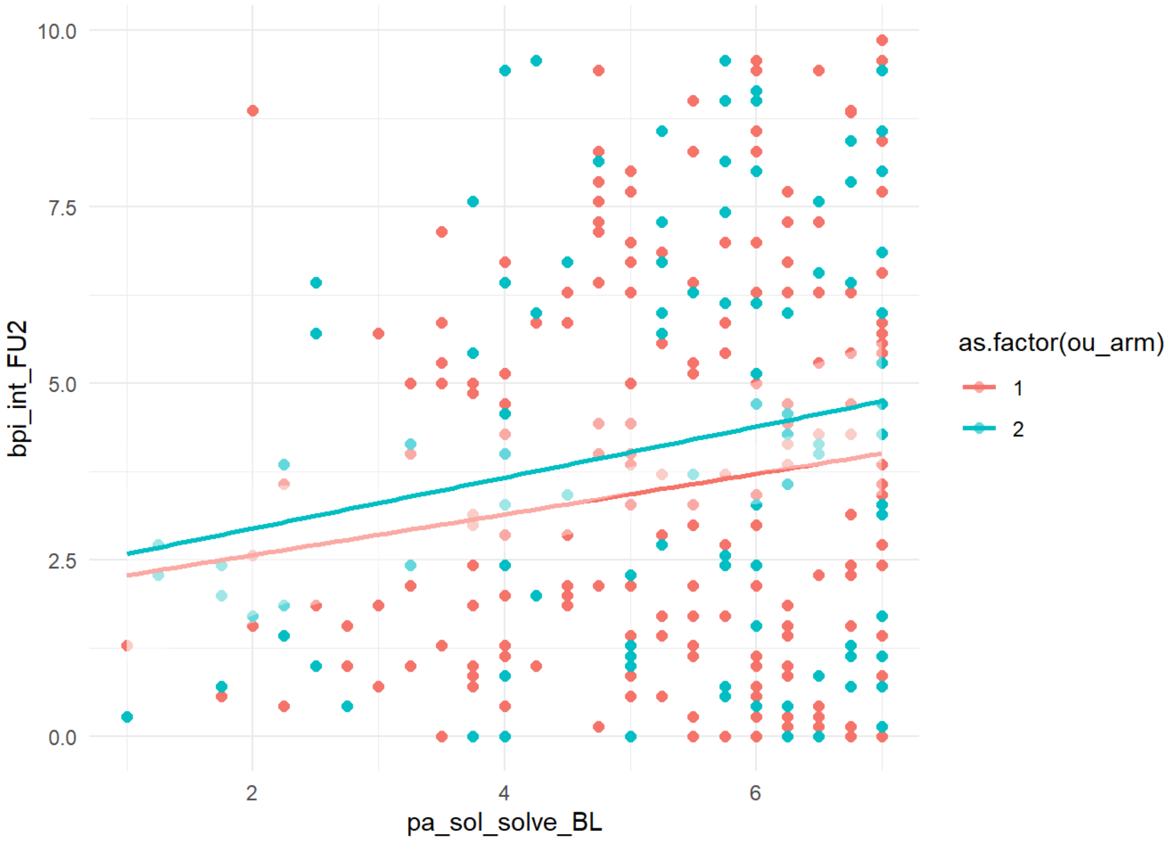


```
ggplot(star_wide, aes(pa_sol_solve_BL, bpi_int_FU2)) +
      geom_point(size = 2, aes()) +
      geom_smooth(aes(), method = "lm", fill = "white")
```

```
## `geom_smooth()` using formula = 'y ~ x'
```

```
## Warning: Removed 50 rows containing non-finite values (`stat_smooth()`).
## Removed 50 rows containing missing values (`geom_point()`).
```

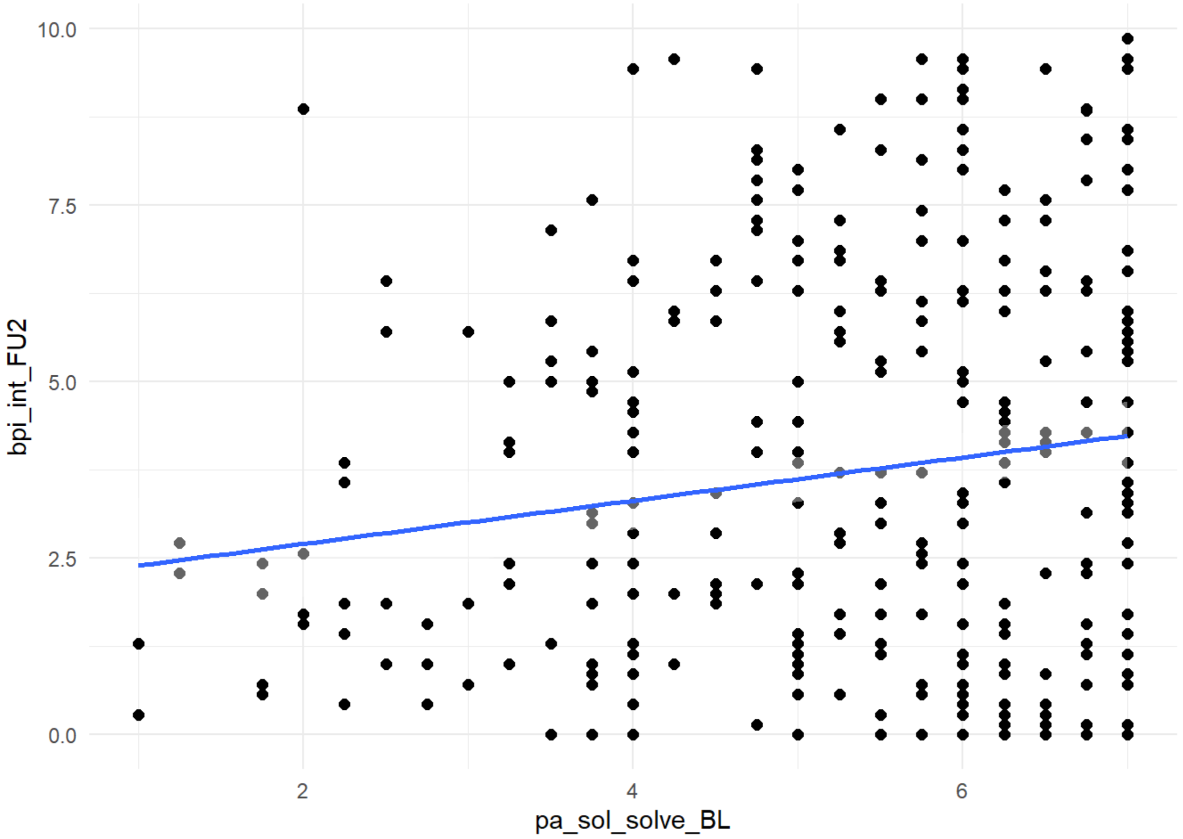


```
model_int_subscales_mod <- lm(bpi_int_FU2 ~ bpi_int_BL +
                         pa_sol_accept_BL * ou_arm +
                         pa_sol_belief_BL * ou_arm +
                         pa_sol_meaning_BL* ou_arm +
                         pa_sol_solve_BL * ou_arm, data = star_wide)


round(Anova(model_int_subscales_mod),2)
```

	Sum Sq <dbl>	Df <dbl>	F value <dbl>	Pr(>F) <dbl>
bpi_int_BL	283.13	1	44.72	0.00
pa_sol_accept_BL	35.11	1	5.55	0.02
ou_arm	29.62	1	4.68	0.03
pa_sol_belief_BL	0.64	1	0.10	0.75
pa_sol_meaning_BL	51.64	1	8.16	0.00
pa_sol_solve_BL	19.38	1	3.06	0.08
pa_sol_accept_BL:ou_arm	0.45	1	0.07	0.79
ou_arm:pa_sol_belief_BL	0.82	1	0.13	0.72
ou_arm:pa_sol_meaning_BL	3.37	1	0.53	0.47
ou_arm:pa_sol_solve_BL	0.73	1	0.12	0.73

1-10 of 11 rows Previous **1** 2 Next

```
summary(model_int_subscales_mod)
```

```
##
## Call:
## lm(formula = bpi_int_FU2 ~ bpi_int_BL + pa_sol_accept_BL * ou_arm +
##     pa_sol_belief_BL * ou_arm + pa_sol_meaning_BL * ou_arm +
##     pa_sol_solve_BL * ou_arm, data = star_wide)
##
## Residuals:
##     Min      1Q  Median      3Q     Max
## -5.4159 -1.8290 -0.2148  1.9539  6.6685
##
## Coefficients:
##                          Estimate Std. Error t value Pr(>|t|)
## (Intercept)               1.95817    2.43293   0.805   0.4215
## bpi_int_BL                0.55097    0.08239   6.687 1.13e-10 ***
## pa_sol_accept_BL          0.15192    0.28427   0.534   0.5934
## ou_arm                   -0.69067    1.63151  -0.423   0.6724
## pa_sol_belief_BL          0.08006    0.34235   0.234   0.8153
## pa_sol_meaning_BL        -0.74624    0.45178  -1.652   0.0996 .
## pa_sol_solve_BL           0.09311    0.31805   0.293   0.7699
## pa_sol_accept_BL:ou_arm   0.05400    0.20258   0.267   0.7900
## ou_arm:pa_sol_belief_BL  -0.08830    0.24580  -0.359   0.7197
## ou_arm:pa_sol_meaning_BL  0.23509    0.32242   0.729   0.4665
## ou_arm:pa_sol_solve_BL    0.07206    0.21247   0.339   0.7347
## ---
## Signif. codes:  0 '***' 0.001 '**' 0.01 '*' 0.05 '.' 0.1 ' ' 1
##
## Residual standard error: 2.516 on 297 degrees of freedom
##   (55 observations deleted due to missingness)
## Multiple R-squared:  0.2353, Adjusted R-squared:  0.2095
## F-statistic: 9.138 on 10 and 297 DF,  p-value: 3.627e-13
```

OKS

```
# Subscales

model_subscales_oks <- lm(oks_FU2 ~ oks_BL +
      pa_sol_accept_BL +
      pa_sol_belief_BL +
      pa_sol_meaning_BL+
      pa_sol_solve_BL, data = star_wide)

round(Anova(model_subscales_oks),2)
```

	Sum Sq <dbl>	Df <dbl>	F value <dbl>	Pr(>F) <dbl>
oks_BL	5503.87	1	75.86	0.00
pa_sol_accept_BL	344.62	1	4.75	0.03
pa_sol_belief_BL	14.47	1	0.20	0.66
pa_sol_meaning_BL	642.20	1	8.85	0.00
pa_sol_solve_BL	39.72	1	0.55	0.46
Residuals	20605.46	284	NA	NA

6 rows

```
summary(model_subscales_oks)
```

```
##
## Call:
## lm(formula = oks_FU2 ~ oks_BL + pa_sol_accept_BL + pa_sol_belief_BL +
##     pa_sol_meaning_BL + pa_sol_solve_BL, data = star_wide)
##
## Residuals:
##      Min       1Q   Median       3Q      Max
## -21.4107  -6.2153  -0.3917   5.7973  24.3812
##
## Coefficients:
##                   Estimate Std. Error t value Pr(>|t|)
## (Intercept)        9.04326    3.21243   2.815  0.00522 **
## oks_BL             0.81767    0.09388   8.710 2.56e-16 ***
## pa_sol_accept_BL  -0.73307    0.33636  -2.179  0.03012 *
## pa_sol_belief_BL  -0.17805    0.39866  -0.447  0.65549
## pa_sol_meaning_BL  1.60496    0.53946   2.975  0.00318 **
## pa_sol_solve_BL   -0.27557    0.37244  -0.740  0.45998
## ---
## Signif. codes:  0 '***' 0.001 '**' 0.01 '*' 0.05 '.' 0.1 ' ' 1
##
## Residual standard error: 8.518 on 284 degrees of freedom
##   (73 observations deleted due to missingness)
## Multiple R-squared:  0.2961, Adjusted R-squared:  0.2838
## F-statistic:  23.9 on 5 and 284 DF,  p-value: < 2.2e-16
```

```
hist(residuals(model_subscales_oks))
```

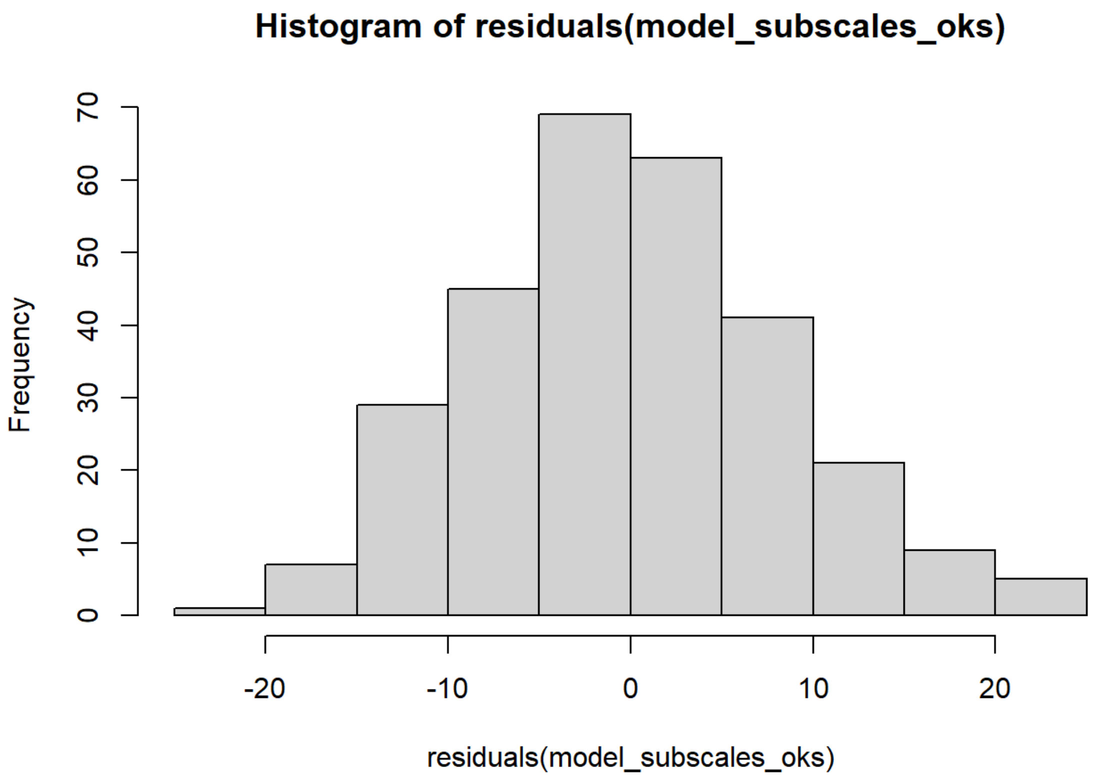


```
ggplot(star_wide, aes(pa_sol_BL, oks_FU2)) +
       geom_smooth(aes(group = ou_arm, color = as.factor(ou_arm)), method = "lm", fill = "whit
e") +
       geom_point(size = 2, aes(color= as.factor(ou_arm)))
```

```
## `geom_smooth()` using formula = 'y ~ x'
```

```
## Warning: Removed 71 rows containing non-finite values (`stat_smooth()`).
```

```
## Warning: Removed 71 rows containing missing values (`geom_point()`).
```

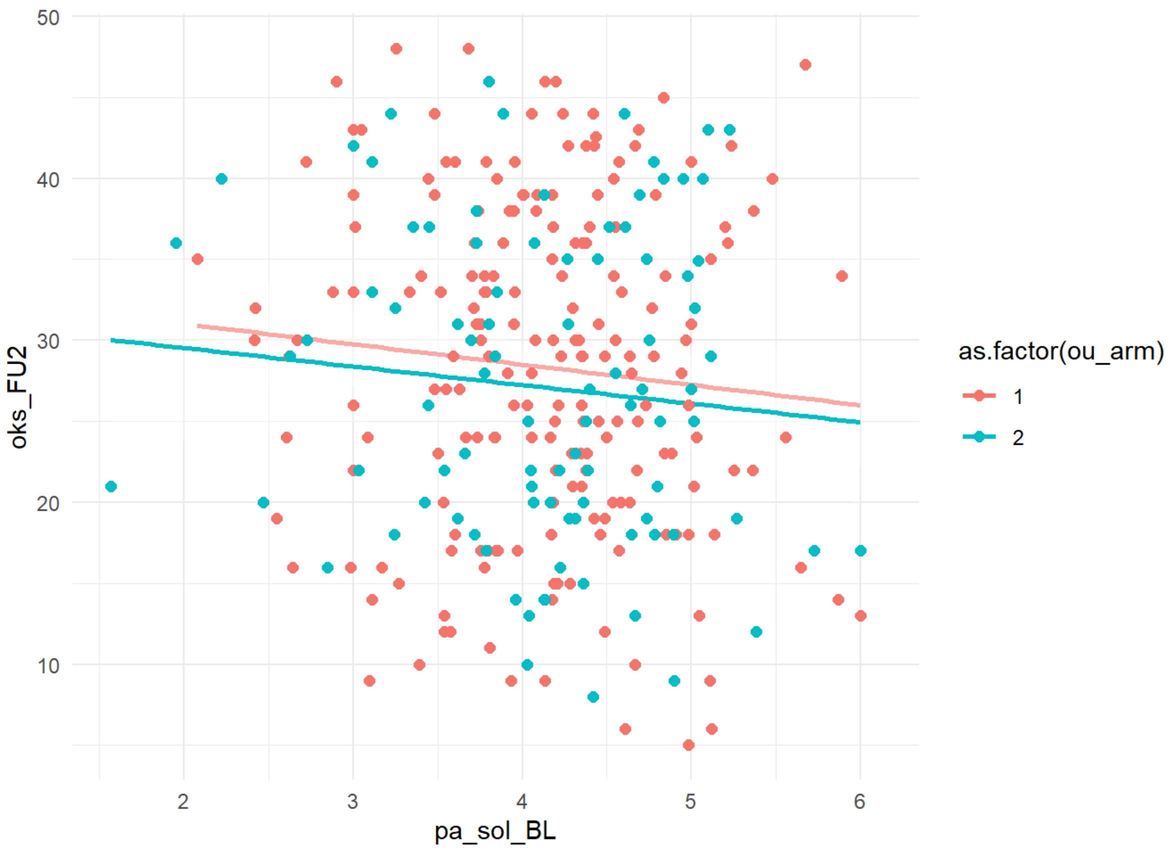


```
ggplot(star_wide, aes(pa_sol_BL, oks_FU2)) +
        geom_smooth(aes(), method = "lm", fill = "white") +
        geom_point(size = 2, aes())
```

```
## `geom_smooth()` using formula = 'y ~ x'
```

```
## Warning: Removed 71 rows containing non-finite values (`stat_smooth()`).
## Removed 71 rows containing missing values (`geom_point()`).
```

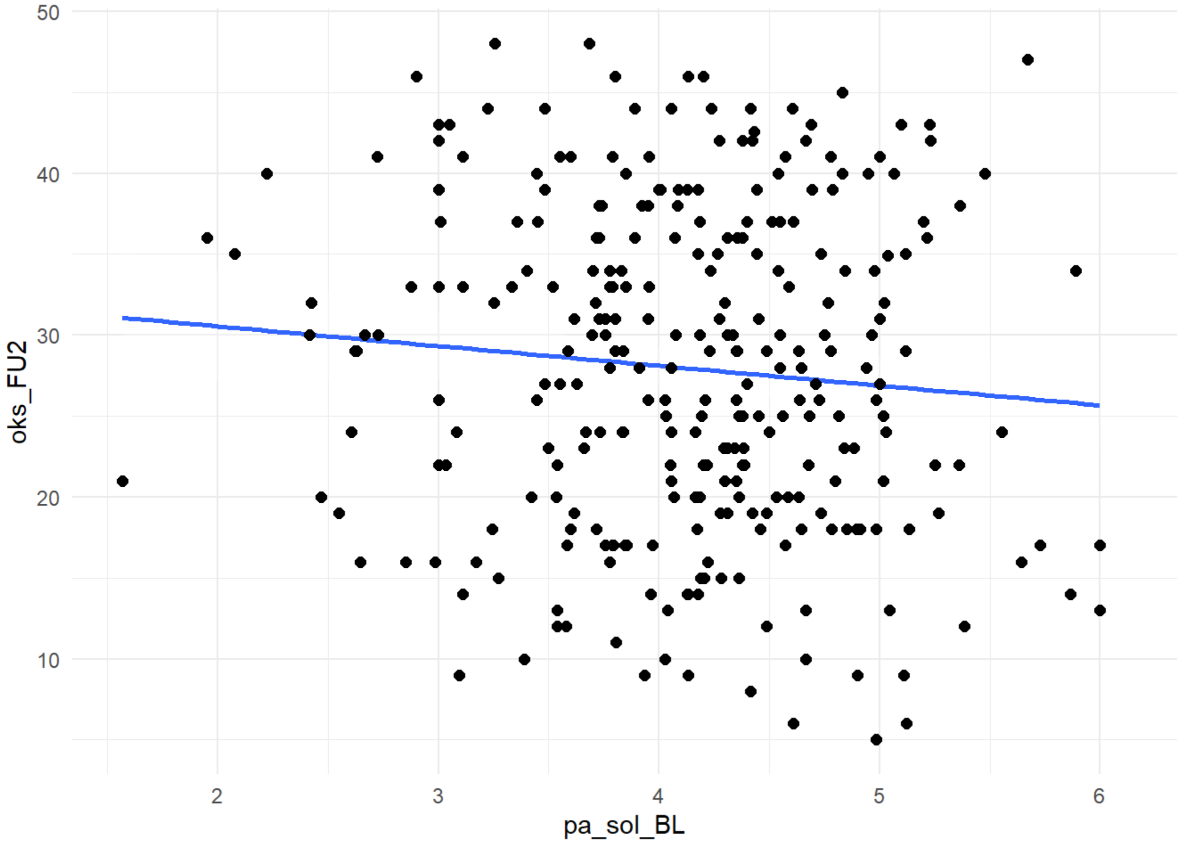


```
ggplot(star_wide, aes(pa_sol_accept_BL, oks_FU2)) +
        geom_smooth(aes(group = ou_arm, color = as.factor(ou_arm)), method = "lm", fill = "whit
e") +
        geom_point(size = 2, aes(color= as.factor(ou_arm)))
```

```
## `geom_smooth()` using formula = 'y ~ x'
```

```
## Warning: Removed 71 rows containing non-finite values (`stat_smooth()`).
## Removed 71 rows containing missing values (`geom_point()`).
```

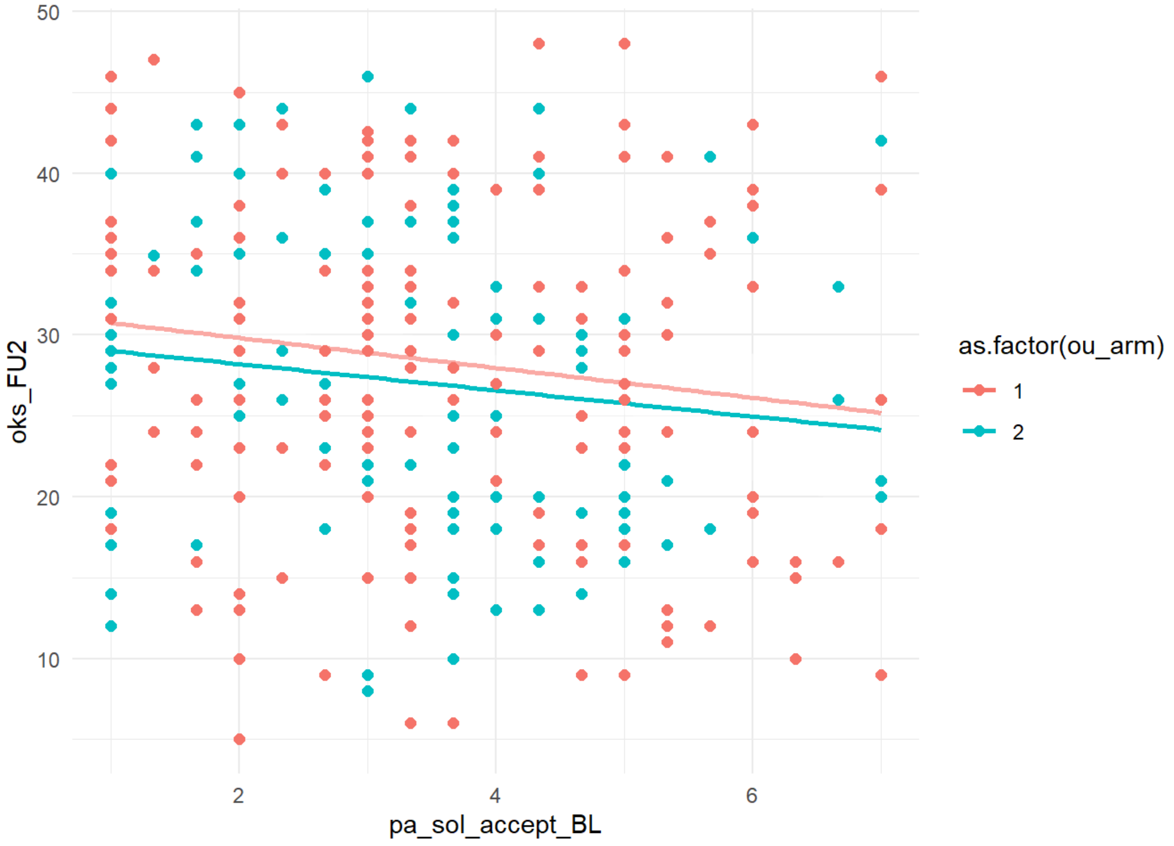


```
ggplot(star_wide, aes(pa_sol_accept_BL, oks_FU2)) +
        geom_smooth(aes(), method = "lm", fill = "white") +
        geom_point(size = 2, aes())
```

```
## `geom_smooth()` using formula = 'y ~ x'
```

```
## Warning: Removed 71 rows containing non-finite values (`stat_smooth()`).
## Removed 71 rows containing missing values (`geom_point()`).
```

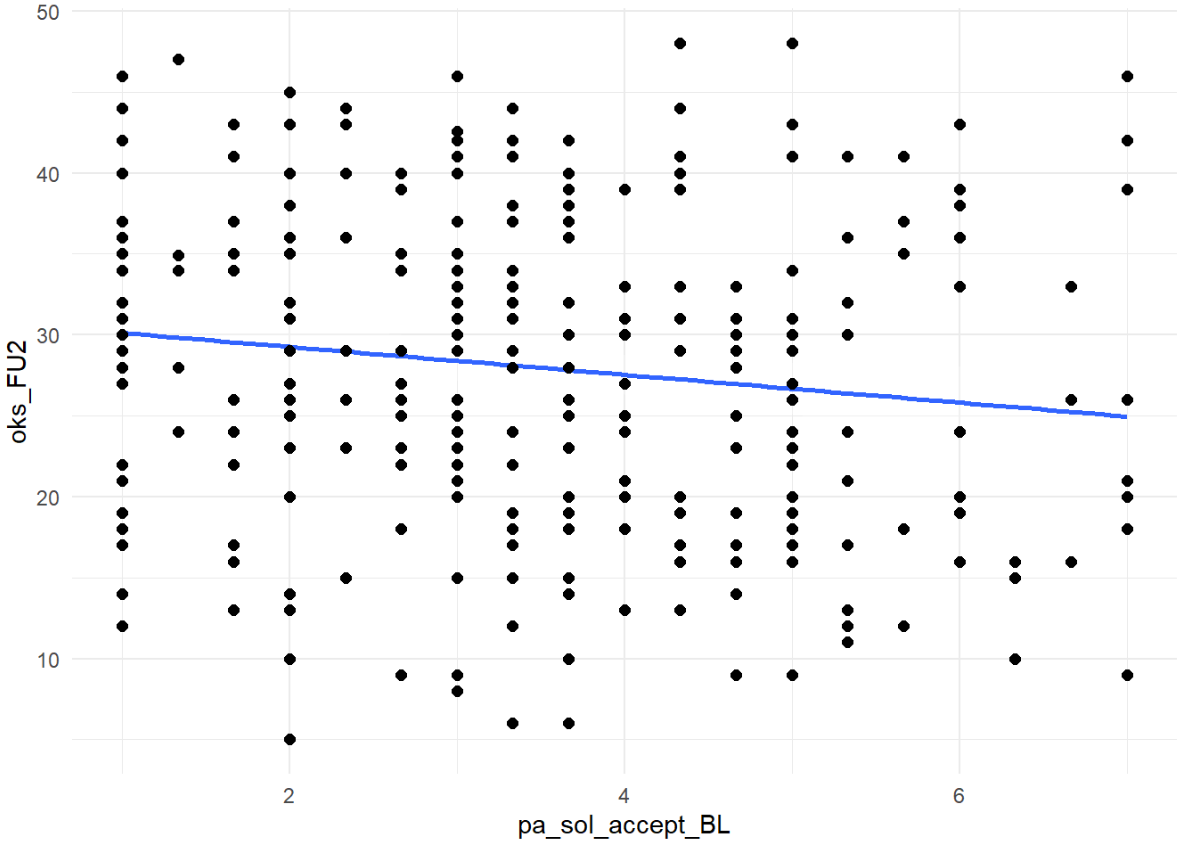

```
ggplot(star_wide, aes(pa_sol_meaning_BL, oks_FU2)) +
        geom_smooth(aes(group = ou_arm, color = as.factor(ou_arm)), method = "lm", fill = "whit
e") +
        geom_point(size = 2, aes(color= as.factor(ou_arm)))
```

```
## `geom_smooth()` using formula = 'y ~ x'
```

```
## Warning: Removed 69 rows containing non-finite values (`stat_smooth()`).
```

```
## Warning: Removed 69 rows containing missing values (`geom_point()`).
```

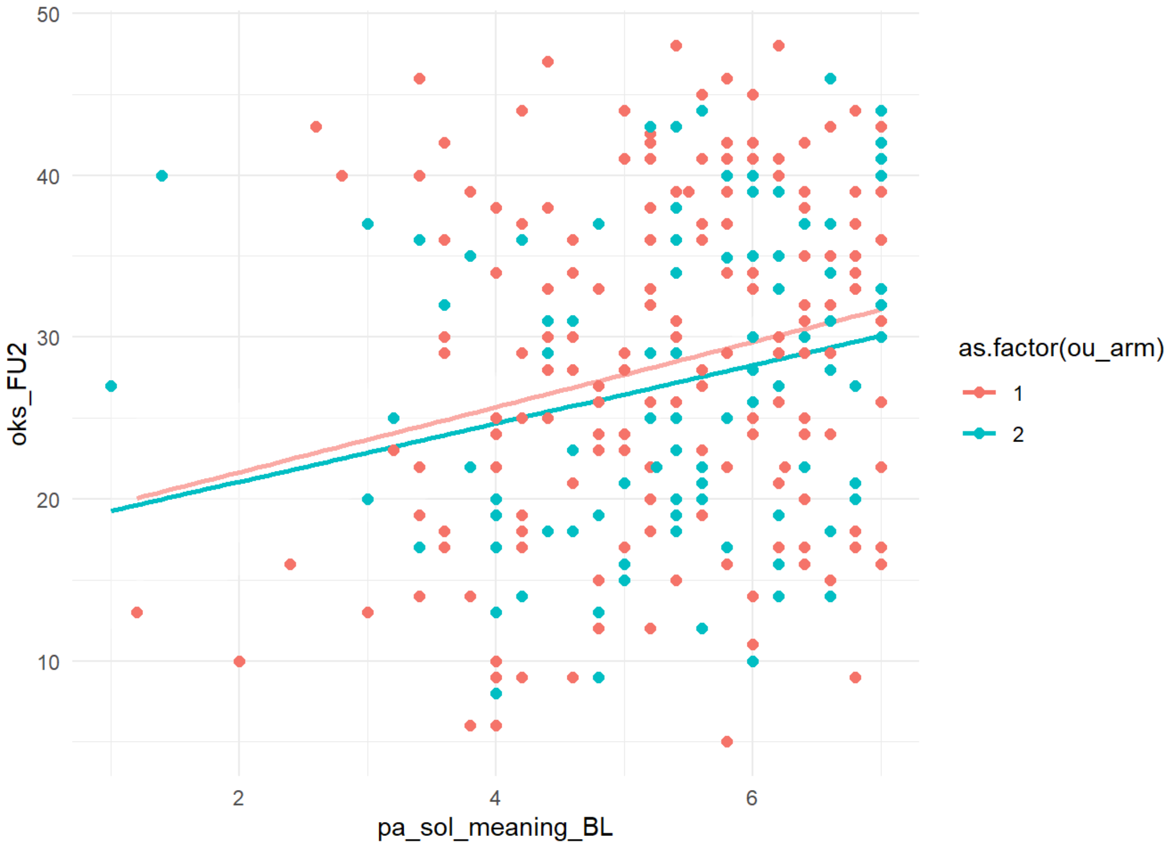


```
ggplot(star_wide, aes(pa_sol_meaning_BL, oks_FU2)) +
        geom_smooth(aes(), method = "lm", fill = "white") +
        geom_point(size = 2, aes())
```

```
## `geom_smooth()` using formula = 'y ~ x'
```

```
## Warning: Removed 69 rows containing non-finite values (`stat_smooth()`).
## Removed 69 rows containing missing values (`geom_point()`).
```

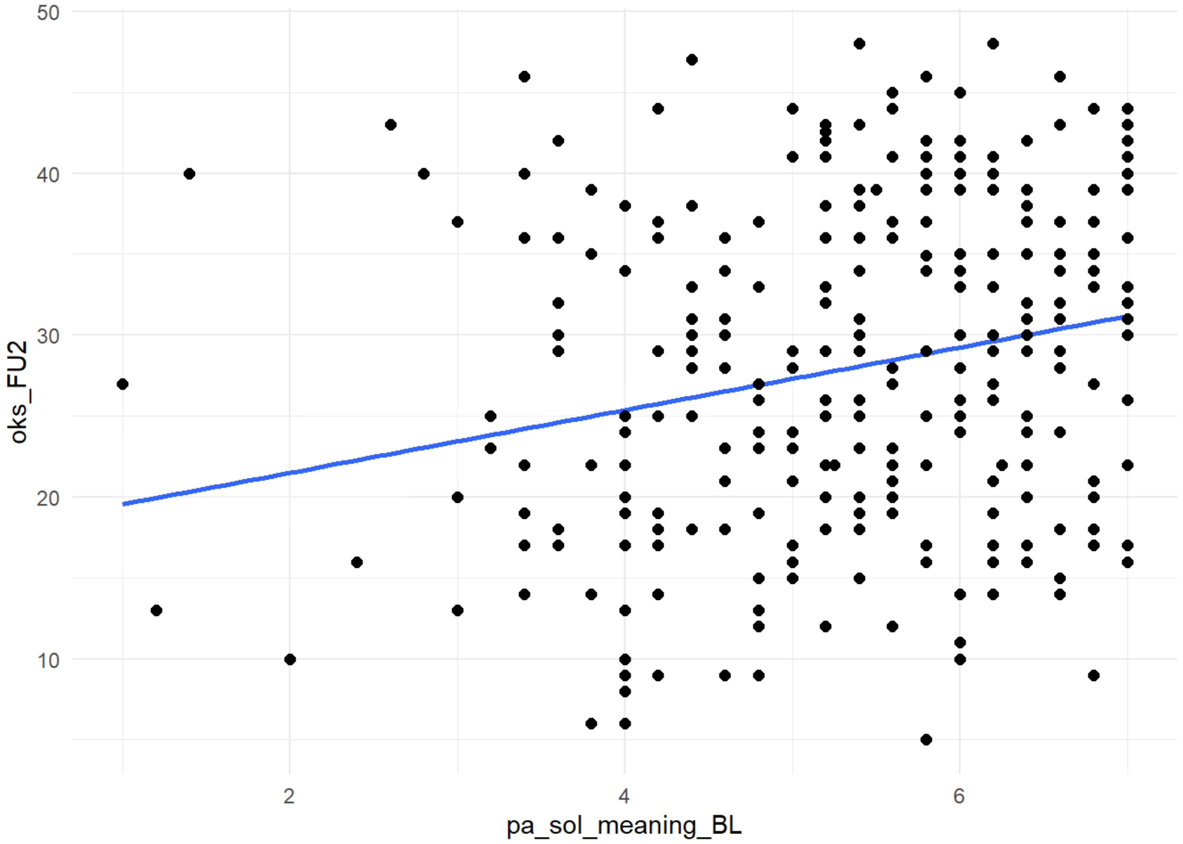


```
ggplot(star_wide, aes(pa_sol_solve_BL, oks_FU2)) +
       geom_smooth(aes(group = ou_arm, color = as.factor(ou_arm)), method = "lm", fill = "whit
e") +
       geom_point(size = 2, aes(color= as.factor(ou_arm)))
```

```
## `geom_smooth()` using formula = 'y ~ x'
```

```
## Warning: Removed 69 rows containing non-finite values (`stat_smooth()`).
## Removed 69 rows containing missing values (`geom_point()`).
```

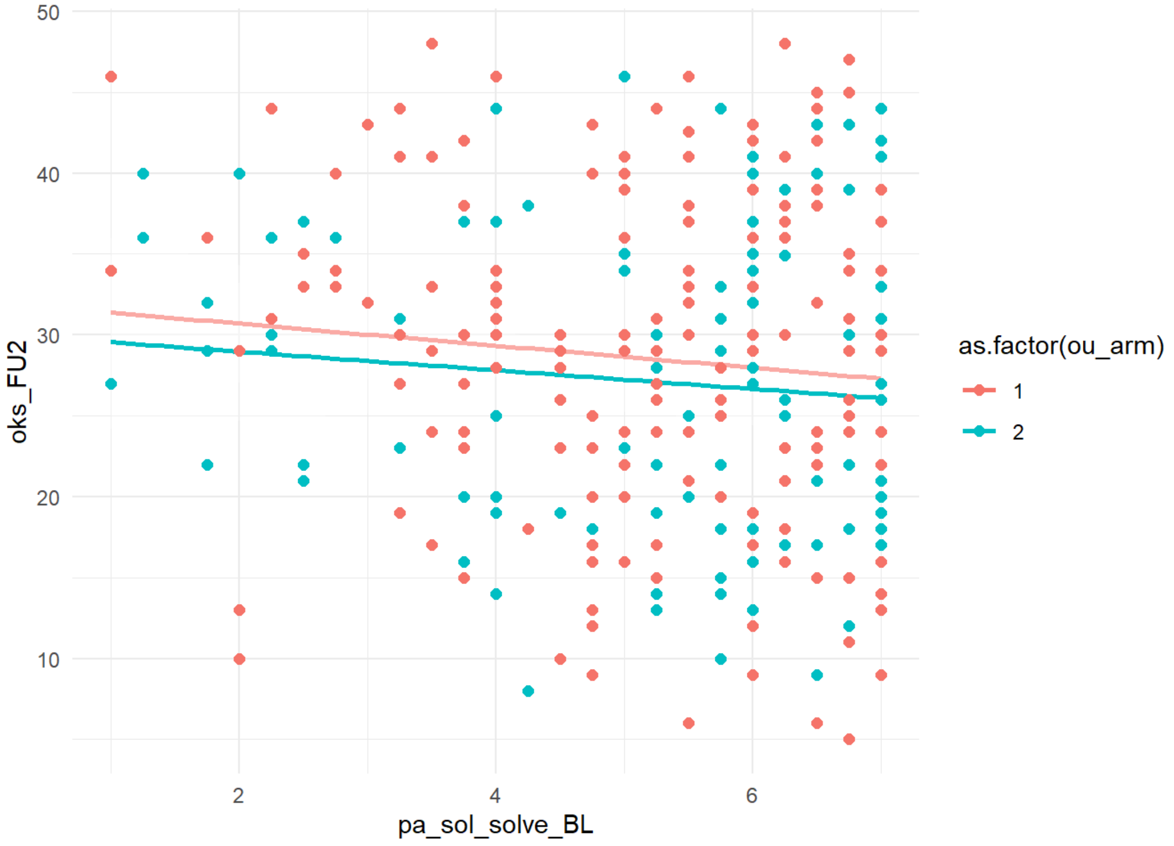


```
ggplot(star_wide, aes(pa_sol_solve_BL, oks_FU2)) +
       geom_smooth(aes(), method = "lm", fill = "white") +
       geom_point(size = 2, aes())
```

```
## `geom_smooth()` using formula = 'y ~ x'
```

```
## Warning: Removed 69 rows containing non-finite values (`stat_smooth()`).
## Removed 69 rows containing missing values (`geom_point()`).
```

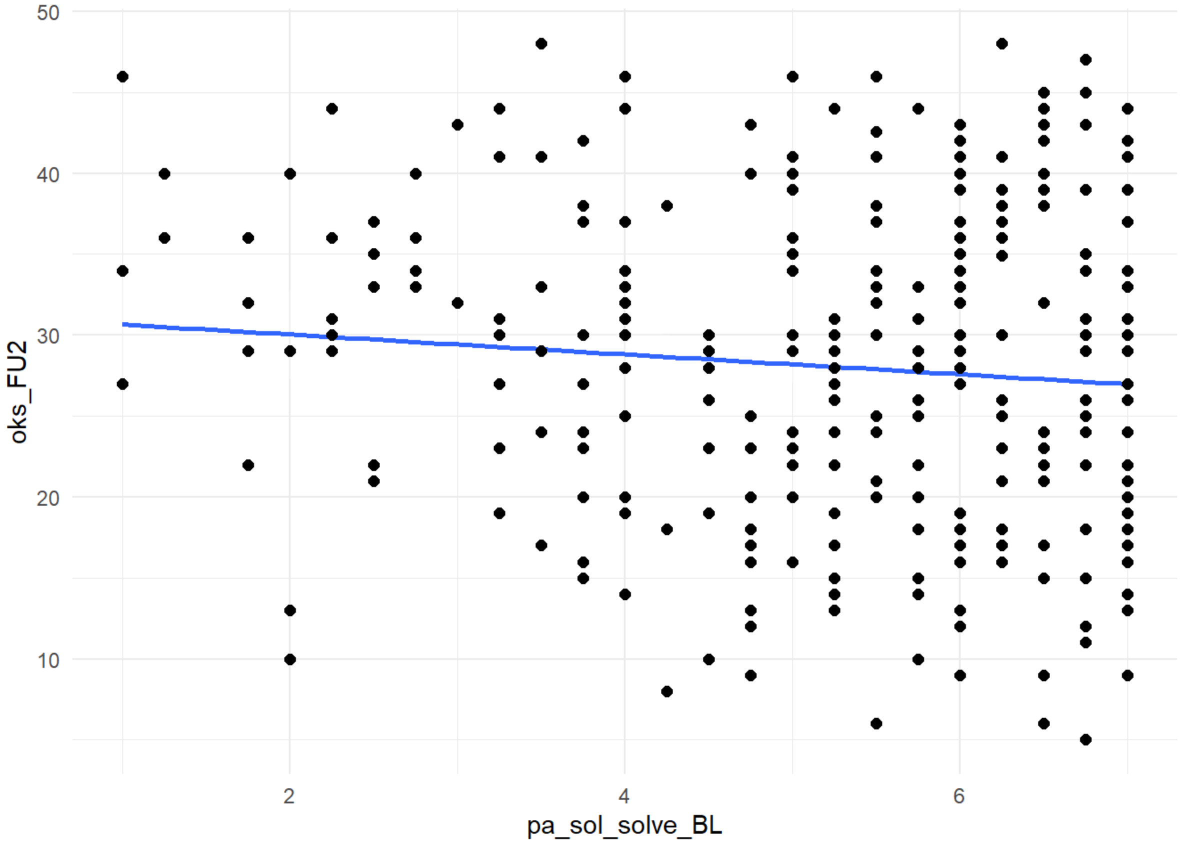

```
model_oks_subscales_mod <- lm(oks_FU2 ~ bpi_int_BL +
                          pa_sol_accept_BL * ou_arm +
                          pa_sol_belief_BL *ou_arm +
                          pa_sol_meaning_BL* ou_arm +
                          pa_sol_solve_BL * ou_arm, data = star_wide)


round(Anova(model_oks_subscales_mod),2)
```

	Sum Sq<br><dbl>	Df<br><dbl>	F value<br><dbl>	Pr(>F)<br><dbl>
bpi_int_BL	3155.31	1	38.87	0.00
pa_sol_accept_BL	724.96	1	8.93	0.00
ou_arm	204.47	1	2.52	0.11
pa_sol_belief_BL	23.54	1	0.29	0.59
pa_sol_meaning_BL	1104.17	1	13.60	0.00
pa_sol_solve_BL	29.06	1	0.36	0.55
pa_sol_accept_BL:ou_arm	1.79	1	0.02	0.88
ou_arm:pa_sol_belief_BL	0.09	1	0.00	0.97
ou_arm:pa_sol_meaning_BL	8.90	1	0.11	0.74
ou_arm:pa_sol_solve_BL	8.34	1	0.10	0.75

1-10 of 11 rows Previous **1** 2 Next

```
summary(model_oks_subscales_mod)
```

```
##
## Call:
## lm(formula = oks_FU2 ~ bpi_int_BL + pa_sol_accept_BL * ou_arm +
##     pa_sol_belief_BL * ou_arm + pa_sol_meaning_BL * ou_arm +
##     pa_sol_solve_BL * ou_arm, data = star_wide)
##
## Residuals:
##      Min       1Q   Median       3Q      Max
## -22.2042  -7.0682  -0.5558   6.3799  22.2227
##
## Coefficients:
##                          Estimate Std. Error t value Pr(>|t|)
## (Intercept)              38.36172    8.94147   4.290 2.46e-05 ***
## bpi_int_BL               -1.89814    0.30446  -6.234 1.67e-09 ***
## pa_sol_accept_BL         -1.20924    1.06430  -1.136    0.257
## ou_arm                   -2.85486    5.99988  -0.476    0.635
## pa_sol_belief_BL         -0.18808    1.28231  -0.147    0.883
## pa_sol_meaning_BL         1.56704    1.67029   0.938    0.349
## pa_sol_solve_BL           0.10823    1.17388   0.092    0.927
## pa_sol_accept_BL:ou_arm   0.11242    0.75731   0.148    0.882
## ou_arm:pa_sol_belief_BL  -0.02986    0.92087  -0.032    0.974
## ou_arm:pa_sol_meaning_BL  0.39457    1.19174   0.331    0.741
## ou_arm:pa_sol_solve_BL   -0.25260    0.78789  -0.321    0.749
## ---
## Signif. codes:  0 '***' 0.001 '**' 0.01 '*' 0.05 '.' 0.1 ' ' 1
##
## Residual standard error: 9.01 on 279 degrees of freedom
##   (73 observations deleted due to missingness)
## Multiple R-squared:  0.2263, Adjusted R-squared:  0.1986
## F-statistic: 8.162 on 10 and 279 DF,  p-value: 1.395e-11
```
